# Supplementary material for: 3D Smith charts scattering parameters frequency-dependent orientation analysis and complex-scalar multi-parameter characterization applied to Peano reconfigurable vanadium dioxide inductors
Source: Sci Rep. 2019 Dec 4;9:18346. doi: 10.1038/s41598-019-54600-5 (PMC6892935; doi:10.1038/s41598-019-54600-5)
Supplement: Supplementary file 1 — Article file in black font [file 41598_2019_54600_MOESM1_ESM.docx]

**3D Smith charts scattering parameters frequency-dependent orientation analysis and complex-scalar multi-parameter characterization applied to Peano reconfigurable vanadium dioxide inductors**

Andrei A. Muller^1*^, Alin Moldoveanu^2^, Victor Asavei^2^, Riyaz A. Khadar^3^, Esther Sanabria-Codesal^4^, Anna Krammer^5^, Montserrat Fernandez-Bolaños^1^, Matteo Cavalleri^1^, Junrui Zhang^1^, Emanuele Casu^1^, Andreas Schuler^5^, Adrian M. Ionescu^1^

^1^ Nanoelectronic Devices Laboratory (NanoLab), École Polytechnique Fédérale de Lausanne (EPFL), 1015, Lausanne, Switzerland,

^2^ Department of Computer Science and Engineering, Faculty of Automatic Control and Computers,

University Politehnica of Bucharest, 060042 Bucharest, Romania,

^3^ Powerlab, École Polytechnique Fédérale de Lausanne (EPFL), 1015, Lausanne, Switzerland,

^4^ Departamento de Matemática Aplicada, Universitat Politècnica de València ,46022 Valencia, Spain,

^5^ Solar Energy and Building Physics Laboratory (LESO-PB), École Polytechnique Fédérale de Lausanne (EPFL), 1015, Lausanne, Switzerland.

^*^Correspondence to: [andrei.muller@epfl.ch](mailto:andrei.muller@epfl.ch)

Recently, the field of Metal-Insulator-Transition (MIT) materials has emerged as an unconventional solution for novel energy efficient electronic functions, such as steep slope subthermionic switches, neuromorphic hardware, reconfigurable radiofrequency functions, new types of sensors, terahertz and optoelectronic devices. Employing radiofrequency (RF) electronic circuits with a MIT material like vanadium Dioxide, VO_2_, requires appropriate characterization tools and fabrication processes.

In this work, we develop and use 3D Smith charts for devices and circuits having complex frequency dependences, like the ones resulting using MIT materials. The novel foundation of a 3D Smith chart involves here the geometrical fundamental notions of oriented curvature and variable homothety in order to clarify first theoretical inconsistencies in Foster and Non Foster circuits, where the driving point impedances exhibit mixed clockwise and counter-clockwise frequency dependent (oriented) paths on the Smith chart as frequency increases. We show here the unique visualization capability of a 3D Smith chart, which allows to quantify orientation over variable frequency.

The new 3D Smith chart is applied as a joint complex-scalar 3D multi-parameter modelling and characterization environment for reconfigurable RF design exploiting Metal-Insulator-Transition (MIT) materials. We report fabricated inductors with record quality factors using VO_2_ phase transition to program multiple tuning states, operating in the range 4 GHz to 10 GHz.

The Smith chart, invented in 1939^1^, is a graphical tool widely used in various fields of electrical engineering and applied physics when dealing with frequency dependent reflection coefficients or impedances. The Smith chart is extensively employed in the design/measurement stage of a large variety of circuits, from metasurfaces^2^ to coils^3^ (Supplementary Fig. 3 in^3^) or scanning microwave microscopy^4^, while being mostly present in microwave-terahertz frequency region in the design and characterization of antennas^5^, transmission lines^6-7^, power amplifiers^8^, filters^9^ or acoustic resonators^10^. The 3D Smith chart proposed in^11^ generalizes the Smith chart (which is limited within the unit circle to circuits with reflection coefficients $(\Gamma=\Gamma_{r}+j\Gamma_{i})$ magnitudes smaller than unity Fig. 1a) onto the Riemann sphere in order to make it usable for all circuits (Fig. 1b and Fig. 1c) further conveying the theoretical support and advance for an intuitive spherical drawing for which the first insights have been presented in^12^. The next developments of the 3D Smith chart^13-14^ propose a Java tool with the 3D Smith chart which additionally displays the group delay and amplifier stability circles too.

An essential drawback of the Smith chart and previous 3D Smith chart representations^1-14^ is the lack of visualization of the variable parameter (frequency), thus the orientation changes and dynamics of the scattering parameters (S) frequency dependency being impossible to be pictured. Although many circuits exhibit a clockwise orientation of their driving point impedances and reflection parameters curves as frequency increases^2-5,9^, (^6,10^ – unspecified), the absence of a clockwise motion (i.e. discontinuity points or counter-clockwise motion) was often reported leading to diverse interpretations. In active devices (transistors), as for example in^15,16^ it is referred as “kink-phenomenon”, in lossless (purely reactive) non-Foster circuits (such as negative capacitors and inductors)^17-20^, as an intrinsic phenomenon. Unfortunately, this counter-clockwise dynamics phenomenon recurrent existence in lossy circuits with non-Foster elements^19,21-24^, led to some misleading conclusions: in^21-24^ the authors assume that the existence of this phenomenon proves the presence of a non-Foster element, while in^25^ it is stated that passive linear devices cannot exhibit driving point immittances with counter-clockwise frequency dependency on the Smith chart.

Because of their lack in zooming capacity, 2D Smith chart representations may oversee also the changes of orientation occurring for the input impedances and reflection coefficients (while increasingly sweeping the frequency), in simple passive networks too. These reversals occur in networks with only Foster elements too as empirically observed in^26-27^. Otherwise orientation reversal phenomenon which occurs in lossy networks with Foster elements is often overlooked or seen as an interpolation error and its presence is often ignored. Since the paths of the reflection coefficients of distinct circuits may coincide within a specific frequency on the Smith chart (as for negative inductors and positive capacitors and viceversa^17,19,20,26^) a frequency dependency visualization is missing in order to get an insight beyond the reflection coefficients paths, regarding their dynamics within it.

From the (differential) geometrical point of view the input impedance and reflection coefficients are particular cases of parametric curves of the frequency variable for each single RF circuit. Their parametric curves equations describe more than a simple geometrical image (their path on the Smith chart), they also contain the information about their motion on it (as for example: orientation and speed).

For this purpose, we first introduce here the notion of oriented curvature *k*^28^ (in our case, frequency dependent- *k(ω)* where *ω* represents the angular frequency and apply it to the reflection coefficients analysis. We prove the mixed clockwise-counter-clockwise orientation phenomenon in lossy circuits with Foster elements and propose a frequency orientation quantification (while increasing the sweeping range) with a new implementation within the 3D Smith chart tool by using the topology of the Riemann sphere^29-30^. Thus, while (increasingly) sweeping the frequency, a new vision to detect this orientation reversal for both Foster and Non-Foster^17-24,31-33^ elements-based networks is first presented. This phenomenon was impossible to quantify for a 2D parametrical plot such as the Smith chart or basic 3D Smith chart (where only the Riemann sphere surface is used).

Further the 3D Smith chart is additionally exploited and developed here in order to display simultaneously parameters needed in reconfigurable frequency designs while dealing with inductors. Frequency dependent inductances based on the *Y_11_*^34-36^ admittance parameter (shunt models *Lshunt*), series models of inductances *Lseries* based on the *Y_21_*^37-38^ parameter, quality factors^34-39^ (*Q*), self-resonant frequencies, Smith chart information^39^ (*Γ)* need ideally all to be simultaneously modeled and analyzed over a wide frequency range during the designs and characterizing stages of inductors. This leads^34-39^ to a larger number of graphics or to different scaling in order to grasp all parameters of interest. Here all these factors are implemented and analyzed on a single combined mode of display using the 3D Smith chart topology and different perspectives.

The newly developed capabilities are particularly suited to explore reconfigurable microwave characterizations, here with reconfigurable CMOS-compatible inductors equivalent circuit modelling for microwaves frequencies using a phase change (PC) material like vanadium Dioxide (VO_2_)^37-38, 40-49^ for tuning the values of inductance. Indeed, it is known that VO_2_ behaves like an insulator under its phase transition temperature Tc = 68 °C (or higher when doped^42^) with monoclinic crystal structure^40^ while deposited on SiO_2_/Si substrates. Because of its ease of integration, reversible insulator to metal transition (IMT), low transition temperature and fast switching time, the employment of VO_2_ as a reconfigurable radio frequency (RF) material has been just recently investigated for a variety of RF-reconfigurable devices^37-38,40,44,45^. Still, much of existing studies are carried out in the frequency range of terahertz or far-infrared^41,43,46,48^, leaving (RF) VO_2_ a largely uncharted area for exploration in development. The conductivity levels of VO_2_ in its insulating (off) state and in its conductive (on) state vary over a wide range depending on the substrate^37-38,40-49^ causing limitations in the RF devices performances (being below 50,000S/m for SiO_2_/Si depositions in the on state).

The on state (limited) conductivity levels of VO_2_ restricted the maximum quality factors (*Q_max_on_*) of the reconfigurable inductors fabricated with this PC material to sub-unitary values^38^, or to values below three in^37^ for CMOS compatible processes on SiO_2_/Si substrates.

Here, after evaluating the VO_2_ conductive/insulating properties, using the new implemented multi-parameter displays, we design and fabricate a new type of SiO_2_/Si CMOS compatible reconfigurable inductors with VO_2_ switches based on Peano curves^50^ and extract their equivalent circuit while analyzing their behavior in a complex-scalar 3D Smith chart-based environment. The aim was to improve the performances obtained in^37^ on SiO_2_/Si substrates in terms of: *Q_max_on_* tuning range and *Q_max_on_/ Q_max_off_* ratio (where Q*_max_off_* denotes the maximum quality factor in the insulating phase of the VO_2_).

The experimentally fabricated and measured reconfigurable inductors improve by 2.33 times the *Q_max_on_* values previously reported in^37^ for the VO_2_ based inductors fabricated within the same CMOS technology on SiO_2_/Si substrates (and by orders of magnitude in respect to^38^), while also increasing the number of reconfiguring states too (from two in ^37^ to three here). Further due to their original geometry the inductors exhibit a tuning range of 77% (improving the 55% in^37^ or 32% in^38^) and have a *Q_max_on_*/ *Q_max_off_* ratio of 0.87 unlike 0.27 in our previous work^37^. It is worth pointing out that the inductor while facing the limited conductivity levels of VO_2_ on SiO_2_/Si substrates reveals a 2.33 better *Q_max_on_* value in in respect to other VO_2_ based reconfigurable inductors such as the SiO_2_/Sapphire inductors reported in^51^ where the conductivity levels of VO_2_ exceed 300.000S/m.

**Oriented curvature of input impedances, reflection coefficients, slope of reactance and 3D Smith chart implementation of frequency dependency orientation**

Based on *k(ω)*^28^ described in detail within the Supplementary Material, we show that the changes in sign of the reactance frequency derivative do not always imply changes in orientation neither for the input impedance nor for the reflection coefficient of 1- port networks (when losses occur). We provide the conditions and equations under which one may have the same orientation (more details in Supplementary Section 1) for both reflection coefficient and input impedances. The lossless (reactive) cases (purely Foster^17-18,27^ and non-Foster^17-20^) become particular cases where the reflection coefficients are direct inversive (Mobius) and indirect inversive transformations^30^ of the oriented imaginary axes of the impedance plane. The clockwise and counter-clockwise motions on circles are a consequence of the reactance slope and sign in the lossless cases.

By introducing the geometrical notion of oriented curvature in this field we prove that the assumptions made by other authors^21-25^ may not apply (see Supplementary Section 1). Further, as seen also for the input impedance of an antenna in^27^, the negative frequency derivative of the reactance of a lossy 1-port network does not imply counter-clockwise motions in the case of lossy 1-port networks. The paths of both 1-port and 2-port networks (such as in Fig. 1) become simple consequences of the magnitude and sign changes of *k(ω) (sgn(k(ω)):*

For any parametric curve $C\left( j\omega\right)$ (1) (a) (all reflection coefficients and input impedances are particular cases of frequency dependent parametric curves) its corresponding *k(ω)*is given by (1) (b).

$C\left( j\omega\right)=$ $a\left( \omega\right)+jb\left( \omega\right)$ (a)  *k(ω)*= $\frac{-b^{'}(\omega)a^{''}(\omega)+a^{'}\left( \omega\right)b^{''}(\omega)}{{({a^{'}(\omega)}^{2}+{b^{'}(\omega)}^{2})}^{3/2}}= \frac{\left| \begin{matrix} a'(\omega) & b'(\omega) \\ a''(\omega) & b''(\omega) \end{matrix} \right|}{{({a^{'}(\omega)}^{2}+{b^{'}(\omega)}^{2})}^{3/2}}$ (b) (1)

Fig. 2 illustrates the concept of oriented curvature along several frequency dependent curves.

Consider now a 1-port network terminated on a resistive load *r*. The input impedance is given by (2), where $r_{m}\left( \omega\right)$ denotes its resistive part and $x_{m}\left( \omega\right)$ its reactive part, while its reflection coefficient is given by (3). Computing the oriented curvature values for both of them (Supplementary Section 1) we get $k_{zm}\left( \omega\right)$ (the oriented curvature of the input impedance) and $k_{\Gamma_{1zm}}\left( \omega\right)$ (the oriented curvature of the 1-port reflection coefficient) as (4) and (5).

$z_{m}\left( j\omega\right)=$ $r_{m}\left( \omega\right)+jx_{m}\left( \omega\right)$ (2)

$\Gamma_{1zm}\left( j\omega\right)=\frac{z_{m}\left( j\omega\right)/r-1}{z_{m}\left( j\omega\right)/r+1}$ (3)

$k_{zm}\left( \omega\right)$ $=\frac{{{r_{m}}^{'}\left( \omega\right)}^{2}\left( \frac{{x_{m}}^{'}\left( \omega\right)}{{r_{m}}^{'}\left( \omega\right)} \right)^{'}}{{({{r_{m}}^{'}\left( \omega\right)}^{2}+{{x_{m}}^{'}\left( \omega\right)}^{2})}^{3/2}}$ (4)

$k_{\Gamma_{1zm}}\left( \omega\right)=\frac{{{{r_{m}}^{'}\left( \omega\right)}^{2}((r+r_{m}\left( \omega\right))}^{2}+{x_{m}\left( \omega\right)}^{2})\left( \frac{{x_{m}}^{'}\left( \omega\right)}{{r_{m}}^{'}\left( \omega\right)} \right)^{'}+2 {x_{m}\left( \omega\right)}^{2}({{r_{m}}^{'}\left( \omega\right)}^{2}+{{x_{m}}^{'}\left( \omega\right)}^{2})\left( \frac{r+r_{m}\left( \omega\right)}{x_{m}\left( \omega\right)} \right)^{'}}{2 r {({{r_{m}}^{'}\left( \omega\right)}^{2}+{{x_{m}}^{'}\left( \omega\right)}^{2})}^{3/2}}$ (5)

Denoting with $x_{mF}\left( \omega\right)$ and $B_{mF}\left( \omega\right)$ the reactance and susceptance of purely Foster elements and with $x_{mNF}\left( \omega\right)$ and $B_{mNF}\left( \omega\right)$ the ones for Non Foster elements we obtain: for Foster networks $r_{m}\left( \omega\right)$ =0 while $\frac{dx_{mF}\left( \omega\right)}{d\omega}>0$and $\frac{dB_{mF}\left( \omega\right)}{d\omega}>0$ and using (3)-(4) we get the input impedance and 1 –port reflection coefficient curvatures for them as:$k_{Fzm}\left( \omega\right)$= 0 and $k_{\Gamma_{1zmF}}\left( \omega\right)$ $=-1$ (Supplementary Section 1). For Non-Foster networks $r_{m}\left( \omega\right)$ = 0 too, while $\frac{dx_{mNF}\left( \omega\right)}{d\omega}<0 and \frac{dB_{mNF}\left( \omega\right)}{d\omega}<0$ hold thus via (4) and (5) we get the input impedance and 1 –port reflection coefficient curvatures for them as $k_{NFzm}\left( \omega\right)$= 0 and $k_{\Gamma_{1zmNF}}\left( \omega\right)=1.$

In the case of two port networks with equal port impedances similar computations can be done for purely Foster and non-Foster elements resulting in the corresponding reflection coefficients $\Gamma_{2zmF}$ and $\Gamma_{2zmNF}$ (6) with their corresponding oriented curvatures (computed in Supplementary Section 1) $k_{\Gamma_{2zmF}}\left( \omega\right)$= -2 and $k_{\Gamma_{2zmNF}}\left( \omega\right)$= 2.

$\Gamma_{2zmF}\left( j\omega\right)=\frac{jx_{mF}\left( \omega\right)}{jx_{mF}\left( \omega\right)+2}$ (a) $\Gamma_{2NF}\left( j\omega\right)=\frac{-jx_{F}\left( \omega\right)}{-jx_{F}\left( \omega\right)+2}$ (b) (6)

Their oriented curvature magnitudes explain the reflection coefficients paths on 0.5 radius circles in Fig. 1d and Fig. 1e on the Smith chart and 3D Smith chart (see Supplementary Section 1). Their oriented curvature opposite signs in (6) determines their reversed orientation.

The reflection coefficients of purely reactive 1-port elements are given in Fig. 3a- Fig. 3d (purely reactive Foster and non-Foster circuits in Fig. 3a, lossy circuits with non-Foster elements in Fig. 3c and lossy circuits with Foster elements in Fig. 3d). The results in Fig. 3d show that reflection coefficients and input impedance orientation reversal can occur at lossy 1-port networks containing only Foster elements too.

The results plotted in Fig. 1d and Fig. 1e, Fig. 3b - Fig. 3c show the new 3D Smith chart implementations capable of detecting orientation changes phenomenon.

The main new insight is given by the representation of the frequency parameter over the 3D Smith chart representation of the reflection coefficient $S_{{11}_{3d}}\left( j\omega\right)$ via a variable homothety with its center in the center of the 3D Smith chart: each frequency that corresponds to a point of the 3D Smith chart reflection point of the $S_{{11}_{3d}}\left( j\omega\right)$ curve will be displayed as a segment on the line that passes from the center of the 3D sphere and the point of the 3D Smith chart surface curve of $S_{{11}_{3d}}\left( j\omega\right)$ . The length of the segment will be given by the normalized frequency and the direction will be outwards of the surface of the 3D sphere. Fig 1d displays in the 3D Smith chart surrounding space the counter-clockwise dynamics of the two port negative capacitor reflection coefficient while the clockwise dynamics of the two port reflection coefficient of the positive inductor. In Fig. 1e one may see the clockwise frequency increasing orientation of the reflection coefficient of the positive capacitor and the counter-clockwise orientation of the negative inductor. (Supplementary Section 2 describes the 3D implementation in detail).

The Smith chart plot can detect the magnitude of the curvature $\left| k_{\Gamma2F}\left( \omega\right) \right|$ (which gives the path of the reflection coefficient-curve shape) but cannot perceive its sign which determines its direction; the new 3D Smith chart frequency orientation quantification and visualization implementation (the frequency sweeping is always increasing in our modelling) detects its sign (see additional video) and thus its orientation. In the cases presented in Fig. 1d and Fig. 1e, $\left| k_{\Gamma2F}\left( \omega\right) \right|$ is constant but not zero thus the shape of the curves is a circle. The same happens in Fig. 3a and Fig. 3b. In more complex circuits one does not deal with reflection coefficients curves with constant curvatures anymore, $k_{\Gamma_{1zm}}\left( \omega\right),$ $k_{\Gamma2F}\left( \omega\right)$ alternate in sign and magnitude values exhibiting orientation changes for both Foster and non-Foster circuits as seen in Fig. 3c and Fig. 3d. In Fig. 3d it can be seen that even a network characterized by a positive real function can generate mixed oriented curvature in its input impedance and reflection coefficient. These reversals of orientation may be easy overlooked on the Smith chart if the zooming scales are not properly chosen but using (4) and (5) this is clearly discovered in Fig. 3d within the sign changes of $k_{\Gamma_{1zmF}}\left( \omega\right)$.

**New 3D visualization insights of frequency dependent series and shunt inductances and quality factors**

The S parameters of the inductors are directly converted by the new implementations in the 3D Smith conceptual software tool into the series inductance model $Lseries\left( \omega\right)$^37-38^ and shunt inductance model $Lshunt\left( \omega\right)$^34-36^ using classical conversion techniques of two port parameters (see Supplementary Section 2). The series and shunt inductances values are then normalized to their maximum value over the frequency range of interest and we get the corresponding normalized values ${Lseries}_{N}\left( \omega\right)$ and ${Lshunt}_{N}\left( \omega\right)$. The reflection parameter *S_11_(jω)* of the inductor is then plotted first on the surface of the 3D Smith chart as $S_{{11}_{3d}}\left( j\omega\right)$ using the previously implemented features^11,13^. Then the 3D space surrounding the 3D Smith chart is used by means of a variable homothethy with the homothetic center in the center of the sphere through the $S_{{11}_{3d}}\left( j\omega\right)$parameter of the inductors. The $S_{{11}_{3d}}\left( j\omega\right)$ parameter is sent now here to another point in 3D at a distance corresponding to ${Lseries}_{N} or L{shunt}_{N}$:

${Lseries,shunt}_{3d}\left( \omega\right)= \left( {LseriesN,shunt}_{N}\left( \omega\right)+1 \right)* S_{{11}_{3d}}\left( j\omega\right)$ (7)

$Q\left( \omega\right)$ of the inductors is also computed by the new 3D Smith chart tool implementation using classical conversion formulas (from the S parameters) (see Supplementary Section 2) and normalized to its maximum value over the frequency range of interest obtaining$Q_{N}\left( \omega\right)$. Using the 3D representation of the ${Lseries,shunt}_{3d}\left( \omega\right)$ curves we then use the normal plane of the curves to associate to each (frequency) point of the curves the $Q\left( \omega\right)$ as a cylinder $Q_{3D}\left( \omega\right)$ of variable radius associated to its normalized value$Q_{N}\left( \omega\right).$ In Fig. 4 one may see the new representations done for the novel fabricated Peano inductor whose performances are compared with the spiral inductor in^37^ (the description on the new inductor design will be presented in the following section).

The new implementation allows the concurrent view of complex valued-scalar parameters:$S\left( j\omega\right)$,${Lseries}_{3d}\left( \omega\right)$, ${Lshunt}_{3d}\left( \omega\right)$, $Q_{3D}\left( \omega\right)$ and frequency (not plotted in Fig. 4 since the dynamics of *S_11_(jω)* is clockwise anyway in this case, unlike the cases presented in Fig. 3). The use of different perspectives and the topology of the 3D Smith chart permits one thus to simultaneously analyze complex parameters (Smith chart) and visualize series and shunt inductances and *Qs* all on the same interactive display. This plays an insightful role in investigation for directly understanding multiple phenomenon on a single view. The information contained in Fig. 4a-Fig.4c can be visualized together using three scaling on a common 2D plot, however still without having any information on *S_11_(jω)* of the inductor. In Fig. 4d we may see just $S_{{11}_{3d}}\left( j\omega\right)$ and ${Lseries}_{3d}\left( \omega\right)$, the display contains already more information than in the 2D Fig. 4a, allowing us to understand that the series inductance model is linear for both analyzed inductors and that $S_{{11}_{3d}}\left( j\omega\right)$ is still in the East hemisphere (in the 4 GHz-10 GHz frequency range) (inductive). Additionally, the zeros of the *S_11_(jω)* are strongly related to the zeros of the *Y_11_(jω*) of an inductor and under certain circumstances (in some) identical (no resistive losses in their equivalent Pi model), thus a change of hemisphere of the *S_11_(jω)* is strongly related to the self-resonances of the inductors model implying in most cases that the *Q* fails to be positive anymore (see Supplementary Section 3). In Fig. 4e we can see $S_{{11}_{3d}}\left( j\omega\right)$,${Lseries}_{3d}\left( \omega\right)$ and $Q_{3D}\left( \omega\right)$ for the 4 GHz-10 GHz frequency range, again the information contained offers an insight on three parameters impossible to visualize together in 2D. In Fig. 4f we may see how by increasing the analysis range up to 15 GHz the *Qs* for both inductors become zero, while the $S_{{11}_{3d}}\left( j\omega\right)$ enters the capacitive hemisphere too. It is interesting to notice that the series inductance model stays linear. Using the 3D Smith chart tool (see supplementary video) one may see the exact frequencies at which $S_{{11}_{3d}}\left( j\omega\right)$ changes hemisphere and the frequency for which *Q* becomes zero (the 3D generalized cylinders become curves-their radius becomes zero (at 14.44 GHz for both inductors). In Fig. 4g$S_{{11}_{3d}}\left( j\omega\right)$,${Lshunt}_{3d}\left( \omega\right)$models become negative starting 14.44 GHz for both inductors and thus they enter the interior of the 3D Smith chart from that frequency point. In Fig. 4h we may see $S_{{11}_{3d}}\left( j\omega\right)$,${Lshunt}_{3d}\left( \omega\right)$ and $Q_{3D}\left( \omega\right)$, clearly $Q_{3D}\left( \omega\right)$ becomes zero once ${Lshunt}_{3d}\left( \omega\right)$ enters the 3D Smith chart. In Fig. 4i all these parameters are shown together.

**Peano reconfigurable inductors using VO_2_ switches**

As a case study of the 3D Smith chart and its usefulness for radiofrequency characterization with VO_2_, and with the aim to improve our reported results in^37^ (in terms of *Q_max_on_, Q_max_on_/ Q_max_off_*, tuning states and tuning range) we have first designed, fabricated and then extracted its equivalent circuit a reconfigurable inductor based on the Peano curve of order 2 by means of VO_2_ switches (Fig.5-Fig.8).

The design of the inductor was done using the similar procedures as in^37^, targeting an extracted *Lseries* of around 1.5 nH in the off state of the both switches and of 0.9 nH in the on state of both while reaching around 1.3 nH if only one switch is used, while targeting to maximize the *Q* within 4-10 GHz frequency in the on state of the VO_2_ where conductivity levels prove to be below 50,000 S/m (please see the Fig.9 in the Methods).

Their Peano geometry is presented in Fig. 5. In Fig. 5a one may see the cross-sectional view of the technology used. The inductors were fabricated using standard microelectronic processes starting with a high-resistivity (10000 Ω·cm) 525 μm thick silicon substrate. A 300 nm thick amorphous silicon layer was first deposited to improve radiofrequency performances^52-53^. The substrate was then passivated with 500 nm SiO_2_ deposited by sputtering. 140 nm-thick VO_2_ and films a Pulsed Laser Deposition (PLD). The film was then patterned using photolithography followed by dry etching and Cr (20 nm)/Al (400 nm) bi-layer was deposited to contact the patterned VO_2_ film. This thin contact layer allowed here the realization of smaller than 0.6 µm gaps between the contact pads (unlike 2μm in our previous work^37^ or 1μm in^54^). Additionally, a 2.4µm-thick Al layer was deposited on top of these contact pads by conventional lift-off methods to provide low RF losses (to create the final CPW elements), the photo of the fabricated inductor being shown in Fig. 5b.

VO_2_ limited on state conductivity levels on Si/SiO_2_ substrates constitute a challenge for the reconfigurable VO_2_ RF design^54^, while to reduce its impact, 1μm switches were employed in the fabricated designs^37,54^.

The small length of the VO_2_ switches obtained here, below 0.6 μm, at the limit of photolithography, minimizes the losses while in the on state (measured as 48,000S/m), while their increased width (120 μm) contributes too to this effort (a tilted photo of the switch in Fig. 5b, is in detail in (Supplementary Section 3). Switch photos and current distributions are further shown in Fig. 5c- Fig. 5f.

In order to validate this claim we additionally fabricated inductors with 1.5 μm and 2.3 switches lengths (Fig. 6a-Fig. 6c and visualized their underwhelming performances in Fig. 6c-Fig 6d.

The inductor has been simulated in the Ansys HFSS commercial software tool while visualizing the extracted inductances and *Q* on the new 3D Smith chart tool. The position of the VO_2_ switches was optimized in order to maximize *Q_max_on_, Q_max_on_ / Q_max_off_*, tuning states and tuning range: the final current distribution at 5 GHz being shown as simulated in Fig. 5c (when both switches are off), Fig. 5e when one switch is off and one on and Fig. 5f with both switches on.

The measured inductances and *Qs* plotted in Fig. 4a- Fig. 4b are compared to our previous work^37^ for the on state of the inductor with two switches show (“on” when measured at 100°C): Peano inductors more than double the *Qs* while also dealing with a smaller inductance (0.9 nH unlike 1.35 nH in^37^, while usually the *Q_max_* decrease a lot while using lower inductances^34^) for the 5GHz-10 GHz frequency range. Further the series inductance is stable with an average value of 0.95 nH within the 4GHz-10 GHz frequency range and thus over-performing our previous reported results^37^. In terms of shunt inductance (untreated in^37,38^) the inductor is stable within the 3 GHz-6 GHz frequency range with an average of 1nH as seen in Fig. 4c.

The overall performances of the inductor with two switches on, two switches off and of a fabricated inductor with one switch on are all plotted together in Fig. 5g (series inductances), Fig. 5h *Qs* and Fig. 5i both *Qs* and normalized 3D series inductances and *Qs*. The results show 77% tuning range and *Q_max_on_*/ *Q_max_off_* 0.88 and thus 3.26 times higher than *Q_max_on_* / *Q_max_off_* reported in^37^. The *Q_max_on_* exceeds 7 being comparable with the off state even though the inductance is tuned with 77% down to 0.95 nH.

On the other hand, the frequency dependency of the off state *Q_off_ (ω)* is comparable with the one reported in^37^ (although dealing with a smaller inductance than in^37^). Overall exhibiting a better performance (in terms of *Q_off_ (ω)*) in the low GHz frequency range and a more stable frequency dependency linearity (in terms of series inductance) the values are facing the same trend as in^37^. The maximum value is limited as in^37^ by the CMOS compatible CPW SiO_2_/Si technology used with Al metallization, (Supplementary Section 3).

**Peano reconfigurable inductors modelling and characterization**

The simplified proposed equivalent circuit of the inductor based on the Fig. 5 inductor’s layout, together with switches equivalent circuit models are presented in Fig. 7 a,b,c. The simplified (frequency independent) Pi model proposed in Fig. 7 uses the classical elements present in inductor modelling^38,55^ as described in Fig.7. The only additional element added is *R_p_* - which is modelling the conductive losses in the adjacent turns due to the presence of the VO_2_ and fabrication flow. In the off state the value of *Rp* (instead of tending to ∞) will be affected by the conductive losses in the VO_2_ (as described in Fig. 7) .VO_2_, whose DC conductivity is presented in Fig.9 will have higher conductivity losses in RF ranges^44^, losses which in the off state will contribute to conductive losses between the turns. In the on state on the other hand, the presence of the VO_2_ (see Fig.5 f) on the main current path will influence the series resistive losses *R_s_* in increasing its value. *R_s_* will thus have an additional component in the on state (besides the turns resistance corresponding to the on state current path), component which will be generated by the VO_2_ resistive losses in the on state.

The extraction of the inductor equivalent circuit parameters is done using an original approach, based on common optimization of 3 (complex) *S* parameters (*S_21_(jω)* = *S_12_(jω)*, due to reciprocity) and extracted *Lseries* and *Q*. Thus we impose the measured *S_ij_(jω)* = *S_ij_circuit_(jω)*-where the last denote the equivalent circuit *S* parameters (and i=1,2) and the *Lseries* and *Q* to be equal to the extracted *Lseries_circuit* and *Q_circuit* of the proposed equivalent circuit .It is worth mentioning that the extraction of the equivalent circuit is usually done using just fitting scalar parameters: series or shunt inductance and *Q*- as in^34^(by us),^35^ (supplementary file), or only series inductance^38^. These approaches are however incomplete, since the *Q* model classically used^34-38,^ is neglecting the right arm of the Pi model, being based on *Y_11_*, thus having the second port grounded^56-57^. These *Q* values (from both measured and equivalent circuit extracted models *Q_circuit*) are accurate only if the inductor is used connected to a ground load ^56-57^. The extracted inductance fitting on the other hand is modeling just an element of the equivalent circuit, model based on only one of the *Y_11_* or *Y_21_*^34-38^ parameters, thus the overall *S* parameters may not fit at all even though *Q* and extracted inductance fitting is reached.

Visualizing the fitting of *S_ij_(jω)* and *S_ij_circuit_(jω)* only on the (2D) Smith chart, as in Figs.1d,e and Figs. 3a may lead to possibly inaccurate solutions, since the paths on the Smith chart may coincide for two circuits on a frequency range even though their nature can be different (which determines their frequency parametrization).

Here we take benefit of the new 3D Smith chart implementations and while using the AWR Microwave Office optimization tool, we simultaneously optimize in the extraction process all *S_ij_circuit_(jω),* *Lseries_circuit* and *Q_circuit* while visualizing this complex-scalar process on the 3D Smith chart.

Figs. 8 a and b show the results on a classical 2D scalar plot, while Figs. 8 c and d on the 3D Smith chart plot. The *S_21_circuit_(jω)* parameter fitting is almost perfect (in both states) (all being in the West hemisphere), while the *S_11_circuit_(jω)*-starts diverging slightly from *S_11_(jω)* in phase at the higher frequencies for both on and off states.

Finally, it can be observed in Fig 8.c or 8.d that both off state *S_11_(jω)* and *S_11_circuit_(jω)* change the hemisphere before 10 GHz (i.e. imag *S_11_(jω)* and *S_11_circuit_(jω)*becomes negative), while their *Q* remains positive (the radius of the generalized cylinders being not yet a point), showing the good match (both change hemisphere) but also the limitations of the inductor when used in a different configuration^57^ than grounded. An inductor *S_11_(jω)* parameter should stay in the inductive part of the Smith chart (East hemisphere-3D Smith chart) for the entire frequency range of interest in order to assure its validity of use under any resistive loads conditions.

**Conclusions**

We have first reported new theoretical foundations for a frequency-dependent 3D Smith with 3D visualization methods for the orientation of parametric curves and used them to quantify and understand curvature reversal, while sweeping the frequency, for driving point impedances and reflection coefficients of circuits in the RF frequency bands. Further we have additionally extended the capabilities of the 3D Smith chart tool to simultaneously visualize a variety of frequency dependent scalar-complex valued parameters required in the inductor modelling and thus proposed a unique multi-parameter display. We demonstrated by fabrication and measurements, original Peano reconfigurable inductors by employing the phase change VO_2_ materials in CPW/ CMOS compatible technology on SiO_2._ The reported inductors improve the previously reported state of art in the incipient field of VO_2_ reconfigurable inductors design for the S, C and X bands of the radio frequency spectrum.

**Methods**

**Fabrication**

The devices were fabricated using a high resistivity (10000 Ω∙cm) Si wafer (525 µm) as the starting substrate. A 300 nm-thick amorphous Si was deposited by low pressure chemical vapor deposition (LPCVD), to reduce the losses during measurement. A surface passivation using 500 nm sputtered SiO_2_ was then carried out, followed by deposition of 140 nm (and 170nm)-thick VO_2_ using a Pulsed Laser Deposition (PLD) system. The film was deposited by pulsed lased deposition (PLD) using a Solmates SMP 800 system. The deposition was performed at 400 ºC in oxygen ambient, with a chamber pressure of 0.01 mbar. The ablated V_2_O_5_ target was placed at 60 mm distance from the wafer. Further the deposition, an annealing of 10 min at 475 ºC was performed without breaking the vacuum in the chamber.

The electrical properties of the films were studied from room temperature up to 100 °C by determining their temperature dependent electrical resistivity converted than in conductivity. This was done by standard four-point probe measurements using a semiconductor parameter analyzer (HP 4156C) and a control on the sample temperature up to 100 °C.

The fabrication process for the Peano inductors on the above substrate then commenced with a photolithography step to pattern the VO_2_ followed dry etching to remove the VO_2_ from the unwanted areas. A Cr-20 nm/ Al-400 nm bi-layer metal stack was then deposited by evaporation after a subsequent photolithography step on the patterned VO_2_. This thin metallization made it possible to realize sub-micron gaps (600 nm) which is critical for extracting a good Q in the conductive state of VO_2_ for the inductors. This was followed by deposition of a 2.4 µm-thick Al layer on these contact pads by conventional lithography followed by metal lift-off procedure to form the CPW elements with low RF losses.

**Devices simulation and characterization**

The numerical simulations of the inductor and filters were done in HFSS ANSYS commercial software relying on the finite element method (FEM) to solve Maxwell equations. Considering the full wave electromagnetic simulation technique, we used the modal solution type for the inductors and the terminal solution type for the filter simulations. The conductivity of the Al was decreased to 3.1*10^7S/m and the VO_2_ switches were modeled for the inductors as simple dielectrics with 20S/m losses and a loss tangent of 0 in the off state. In the on state, the VO_2_ switches were simulated as lossy metals of a conductivity of 48,000S/m. Subsequently full inductors and filters models were built in the software according to the actual physical structure fabricated. The Peano shapes were implemented using the Equation Curve facility of the tool, their equations being written parametrically and where needed rotations and reflections were used for the very final shapes.

The devices were measured with the Anrtisu Vector Star VNA in a Cascade Summit prober with controllable chuck temperature who was set to 20^°^C in the “off state” and 100^°^C in the on state. For the 2D graphical interpretation the measured S parameters were converted using the Anritsu Star VNA installed Microwave Office too into the desired parameters analysed. In order to obtain the extracted equivalent circuit parameters circuit we used the Genetic Algorithm of the Anritsu-AWR MW Office installed tool and optimization of 3 complex parameters and *Sij(jω) = Sij_circuit(jω)* and two scalars: *Lseries*= *Lseries_circuit* and *Q*= *Q_circuit* – in the 4 GHz-10 GHz range using 15,000 iterations.

Figure 9 presents schematically the inductors layouts and characterized parameters:

**3D Smith Chart implementation**

The 3D Smith Chart application is developed using the Java programming language and the following libraries and development environment are used:

- 3D rendering: OpenGL through the Java Binding for the Open GL API (JOGL2) library;
- Mathematical operations and complex data representation: The Apache Commons Mathematics Library;
- Development environment: The NetBeans IDE with Beans Binding Library for the implementation of the application GUI and JOGL2 usage.

Further implementation details about the new mode of visualization, new simulation parameters used in the paper and their 3D representation on the Riemann sphere can be found in Supplementary Section 2.

**Mathematical modelling of curvature**

The calculations for the oriented curvature was performed using Mathematica software tool by writing the frequency parametric equations of the curves analyzed.

**References**

1. Smith, P.H. Transmission-line calculator. *Electronics* **12**, 29–31 (1939).
2. Zhu, B.O., Zhao, J., Feng, Y. Active impedance metasurface with full 360° reflection phase tuning. *Sci. Rep.* **3**, 3059 (2013).
3. Thang, B., Sodickson, D.K., Cloos, M.A. A high-impedance detector-array glove for magnetic resonance imaging of the hand. *Nat. Biomed. Eng.* **2**, 570-577 (2018).
4. Tuca, S.-S. et al. Calibrated complex impedance of CHO cells and E. coli bacteria at GHz frequencies using scanning microwave microscopy. *Nanotechnology* **27**, 135702 (2016).
5. Qu L., Zhang, Zhang R., Shin, H., Kim J., Kim, H. Mode-Controlled Wideband Slot-Fed Ground Radiation Antenna Utilizing Metal Loads for Mobile Applications. *IEEE Trans. Antennas Propag*. **65,** 2, 867-872 (2017).
6. Chu H.C, Ma, T. Beamwidth switchable planar microstrip series-fed slot array using reconfigurable synthesized transmission lines. *IEEE Trans. Antennas Propag.* **65**, 7, 3766-3771 (2017).
7. Liu, C. et. al. Long-distance propagation of short-wavelength spin waves, *Nat. Commun.,* **9**, 738 (2018).
8. Liu S., Liu A. A novel design methodology for high-efficiency current-mode and voltage-Mode Class-E Power Amplifiers in Wireless Power Transfer systems. *IEEE Trans. Power Electron.* **32**, 6, 4514-4521 (2017).
9. Ma J., Steer, M. B., Jiang X. An acoustic filter based on layered structure. *Appl. Phys. Lett.* **106**, 111903 (2015).
10. Dubois M.-A., Murait, P. Properties of aluminum nitride thin films for piezoelectric transducers and microwave filter applications. *Appl. Phys. Lett.* **74**, 20, 3032-3034 (1999).
11. Muller, A. A., Soto, P. Dascalu, D, Neculoiu, D. Boria, V. E. A 3d Smith chart based on the Riemann sphere for active and passive microwave circuits. *IEEE Microw. and Wireless Compon. Lett.,* **21**, 6, 286-288 (2011).
12. Zelley Chris, A spherical representation of the Smith chart, *IEEE Microw. Mag.*, **8**, 3, 60-66, (2007).
13. Muller, A., A., Sanabria-Codesal, E., Moldoveanu, A. Asavei, V., Lucyszyn, S. Extended capabilities with group delay and resonator quality factor. *IEEE Trans. Microw. Theory Techn.,* **65**, 1, 10-17 (2017).
14. Muller, A. A., Moldoveanu A., Asavei V., Fleischer C. 3D Smith chart tool (2019) [www.3dsmithchart.com](http://www.3dsmithchart.com).
15. Shan, A., A. et al. Physics-based multi-bias RF large-signal GaN HEMT modeling and parameter extraction flow. *IEEE J. Electron Devices Soc.* **5**, 5, 310-318 (2017).
16. Lu, S.-S., Meng, C., Chen, T.-W. The origin of the kink phenomenon of transistor scattering parameter S_22_. *IEEE Trans. Microw. Theory Techn.*, **49**, 2, 333-340 (2001).
17. Mirzaei, H., Eleftheriades G., V. Realizing non-Foster reactive elements using negative-group-delay networks. *IEEE Trans. Microw. Theory Techn.*, **61**, 12, 4322–4332 (2013).
18. Muller, A.A., Lucyszyn, S., Properties of purely reactive Foster and Non-Foster passive networks. *IET Electron. Lett.,* **51**, 23, 1882-1884 (2015).
19. Jacob, M.M. Non-Foster circuits for high performance antennas: advantages and practical limitations. In *PhD thesis* San Diego UCSD (2016), https://escholarship.org/uc/item/2pq549c3.
20. Sievenpiper, D., Jacob, M., Long, J. Materials and components with a negative frequency derivative of reactance. In UCSD presentation (2017). <https://e3s-center.berkeley.edu/wp-content/uploads/2017/07/PEB2012_7_DSievenpiper_Webfinal.pdf>
21. Tade, O.O., Gardner. P., Hall, S.S. Negative impedance converters for broadband antenna matching. In 2012 *42nd European Microwave Conference* (2012)*,* https://ieeexplore.ieee.org/document/6459295 (2012).
22. Tade, O.O., Gardner. P., Hall, S.S. Broadband matching of small antennas using negative impedance converters. In 2012 *IEEE International Symposium on Antenna and Propagation and USNC-URSI National Radio Science meeting* (2012). *https://ieeexplore.ieee.org/document/6348833*
23. Tade, O.O., Gardner. P., Hall, S.S. Antenna bandwidth broadening with a negative impedance converter. *Int. J. Microwave T*. **5** (3), 249-260 (2013).
24. Hrabar, S., Krois, I. Kiricenko, A., Towards active dispersion less ENZ metamaterial for cloaking applications. *Metamaterials*, **4**, 2-3, 89-97 (2010).
25. Goldstone, L. A Low VSWR matching technique. (Correspondence and author’s reply). *IRE Trans. Microw. Theory Techn*. **5l**, 2, 163 (1957).
26. Munk, B.A. *Metamaterials: Critique and Alternatives*. John Wiley& Sons Inc., New Jersey, USA (2009).
27. Best, S.R., The Foster reactance theorem and quality Factor for antennas. *IEEE Antennas Wireless Propag. Lett.* **3**, 306-309 (2004).
28. Berger, M., *A Panoramic View Of Riemannian Geometry*. Springer, Berlin (2003).
29. Bochert, M. Infinite Regions of Various Geometries. *Bull. Amer. Math. Soc*., 20, 4,185-200 (1914).
30. Brannan, D.A., Esplen, M.F., Gray, J.J., *Geometry*. Cambridge University Press, New York (2007).
31. Shi, T., Tang, M.-C., Wu, Z., Xu, H., Ziolkowski, R.W., Improved signal-to Noise Ratio (SNR), Bandwitdh-enhanced electrically small antenna augmented with Internal non-Foster elements. *IEEE Trans. Antennas Propag*. In press (2019) <https://ieeexplore.ieee.org/stamp/stamp.jsp?tp=&arnumber=8624545&tag=1>
32. Kemp, A.M., et al, A high Q piezoelectric resonator as a portable VLF transmitter. *Nat. Commun.,* **10**, 1715 (2019).
33. Fleury, R., Sounas, D., Alu, A., An invisible acoustic sensor based on parity-time symmetry. *Nat. Commun.,* **6**, 5905 (2015).
34. Leroy, C. et al., High quality factor copper inductors integrated in deep dry-etched quartz substrates. *Microsyst. Technol.*, **13**, 11–12, 1483–1487 (2007)
35. Kang, J. et. al., On-chip intercalated-graphene inductors for next-generation radio frequency electronics. *Nature Electronics*, **1** 46-51(2018).
36. Wainstein, N., Kvatinsky, S. Time-tunable inductors using mermistors. *IEEE Trans. Circuits Syst. I, Reg. papers*, **65**, 5, 1505-1515 (2018).
37. Casu, E.A. et al. A reconfigurable inductor based on Vanadium Dioxide insulator to metal transition. *IEEE Microw. Compon. Lett*. **29**, 9, 795-797 (2018).
38. Wang, S., Wang, W., Shin, E., Quach, Subramanyam, G. Tunable inductors using vanadium dioxide as the control material. *Microw. Opt. Techn. Lett.*, **59**, 5, 1057–1061 (2017).
39. Jung, Y.H. *et al*. High-performance green flexible electronics based on biodegradable cellulose nanofibril paper. *Nat. Commun.* **6**, 7170 (2015).
40. Zhang, G., Ma, H., Lan, C., Gao, G., Zhou, J. Microwave Tunable Metamaterial Based on Semiconductor-to-Metal Phase Transition. *Sci. Rep.* **7**, 577 (2017).
41. Prayakrao, S. et al. Tunable VO_2_/Au hyperbolic metamaterial. *Appl. Phys. Lett.* 109, 061105 (2016).
42. Krammer, A. et al. Elevated transition temperature in Ge doped VO2 thin films. *J. Appl. Phys*. **122**, 045304 (2017).
43. Stinson, H.T. et al. Imaging the nanoscale phase separation in vanadium dioxide thin films at terahertz frequencies, *Nat. Commun.* **9**, 3604 (2018).
44. Emond, N., Henadaoui, A., Delpart, S., Chaker, M.,Wu, K. Theoretical and Experimental Investigation of Thermo-Tunable Metal–Insulator–Vanadium Dioxide Coplanar Waveguide Structure. *IEEE Trans. Microw. Theory Techn.*, **65**, 5, 1443–1455 (2017).
45. Casu, E.A., et al. Vanadium Oxide bandstop tunable filter for Ka frequency bands based on a novel reconfigurable spiral shape defected ground plane CPW. *IEEE Access*, 6, 12206-12212, (2018).
46. Zheng, X., Xiao, Ling, Z. X. A Tunable Hybrid Metamaterial Reflective Polarization Converter Based on Vanadium Oxide Film. *Plasmonics*, **13**(1) 287-291 (2018).
47. Kovacs, G.J, et al. Effect of the substrate on the insulator–metal transition of vanadium dioxide films. *J. Appl. Phys*. 1**09**, 6, 063708 (2011).
48. Vitale, W. A. et al. Modulated scattering technique in the terahertz domain enabled by current actuated vanadium dioxide switches. *Sci. Rep.* 7, 41546 (2017).
49. Vitale, W. A. et al. A Steep-Slope Transistor Combining Phase-Change and Band-to-Band-Tunneling to Achieve a sub-Unity Body Facto, *Sci. Rep.* 7, 355, (2017).
50. Fan. J. A., et al. Fractal design concepts for stretchable electronics. *Nat. Commun.* **5**, no. 3266, (2014).
51. Li, L. Wang, W., Shin, E., Subramananyam, G. Tunable inductors using integrated Vanadium Dioxide phase change thin films. *Advances in Condensed Matter Physics*, **18**, 1-7 (2018).
52. Fernandez-Bolanos, M., Perruisseau-C., Dainesi, P., Ionescu. A.M., RF Mems capacitive switch on semi-suspended CPW using low loss high resistivity silicon substrate. *Microelectron. Eng*., 85, 6, 1039-1042, (2008).
53. Gamble, H.S. et al., Low-Loss CPW lines on surface stabilized high-resistivity silicon. *IEEE Microw. Guided Wave Lett.*, 9, 10, 395-397, (1999).
54. A.A. Muller et al, A novel reconfigurable CMOS compatible Ka band bandstop structure using split-ring resonators and Vanadium Dioxide (VO_2_) phase change switches. In 2019 IEEE MTT-S International Microwave Symposium digest, 865-867.
55. Yue, C.P, Wong, S.S, Physical modeling of spiral inductors on silicon. *IEEE Trans. Electron Dev.* 47, 3*,*560-568 (2000).
56. Horng, T. S., Peng, K. C., Jau, J.K, Tsai, S-parameter formulation of quality factor for a spiral inductor in generalized two-port configuration. In 2003 IEEE MTT-S International Microwave Symposium digest, 255-258.
57. Horng, T. S., Peng, K. C., Jau, J.K, Tsai, Y.S. S-parameter formulation of quality factor for a spiral inductor in generalized two-port configuration. *IEEE Trans. Microw. Theory Techn.*, **51**, 11, 2197–2202 (2003).

**Figure legends**

**Figure 1 | Smith chart limitations and clockwise and counter-clockwise frequency dependency of Foster and non-Foster elements on a newly introduced frequency dependent 3D Smith chart. a,** Smith chart. **b,** Extended Smith chart **c,** 3D Smith chart as in 2018 (without frequency dependency). **d,** 3D Smith chart representation of the two port reflection coefficient and newly implemented frequency dependent 3D Smith chart representation for it. For a capacitor with purely negative capacitance and an inductor with positive inductance their reflection coefficient frequency representation overlaps on the Smith chart for a wide frequency range, their orientation changes cannot be distinguished. On the newly implemented frequency dependent 3D Smith chart (when compared to our previous works in^11,13,14^) one can clearly see their clockwise motion with increasing frequency for the inductor while the counter-clockwise motion for the capacitor with negative capacitance. **e,** 3D Smith chart representation of the reflection coefficient and newly implemented frequency dependent 3D Smith chart for a negative valued inductor and capacitor with positive capacitance. Again, their trace is identical on a Smith chart, their intrinsic opposed frequency dependency cannot be seen. On the 3D Smith chart one can directly see the clockwise motion of the reflection coefficient of the capacitor and the counter-clockwise motion of the reflection coefficient of the negative valued inductor.

***Figure 2 |* Oriented curvature k(ω) and its sign sgn(k(ω)) for various motions on various reflection coefficient or impedance curves *C(jω)*. a,** $k\left( \omega\right)>0$ when $C\left( j\omega\right)$ is counter-clockwise oriented; $k\left( \omega\right)<0$ where $C\left( j\omega\right)$ is clockwise oriented. **b,** $k\left( \omega\right)=0$ for any line shape of $C\left( j\omega\right)$. **c,** When $C\left( j\omega\right)$ has a circle shape of radius 2 and a counter-clockwise motion $k\left( \omega\right)=1/2$. **d**, When $C\left( j\omega\right)$ has a circle shape of radius 1 and a clockwise motion $k\left( \omega\right)=-1.$

**Figure 3 | Reflection coefficient orientation changes and the sign of the oriented curvature for different circuits. a,** Smith chart representation of the reflection coefficient for a 1-port negative capacitance (purely non-Foster circuit) and a positive inductance (purely Foster). For a capacitor with purely negative capacitance and an inductor with positive inductance their reflection coefficients $\Gamma_{1zm}(j\omega)$ overlap on the Smith chart on a wide frequency range. Their opposite orientation is given by the different sign of their oriented curvature $k_{\Gamma_{1zm}}\left( \omega\right)$. Their same path is given by the same absolute value of their oriented curvature. **b,** On the newly implemented frequency dependent 3D Smith chart one can see the clockwise motion with increasing frequency for the inductor with positive inductance and the counter-clockwise motion for the negative valued capacitor, the motion is on the contour of the equatorial plane (lossless circuits). **c,** Mixed motion for a fabricated circuit containing non-Foster (lossy elements). **d,** Mixed clockwise and counter-clockwise motion of the reflection coefficient of a passive lossy network described by the positive real function $z_{m}\left( s \right)$ with the 1 port reflection coefficient (for s=jω) $\Gamma_{1zm}\left( j\omega\right)$. The reflection coefficient has a clockwise orientation from -2<ω<-0.28 and for 0.28<ω<2, while counter-clockwise orientation for -0.28<ω<0.28. The sign changes of its 1-port reflection coefficient curvature $k_{\Gamma_{1zm}}\left( \omega\right)$(5) generates the changes of orientation of its path on the Smith chart. It is interesting to notice that mixed motion can exist on limited bandwidth also for lossy circuits with only Foster elements and thus that the counter-clockwise motion is by no means a prove of an existence of a non-Foster element in the network. A more detailed description on oriented curvature and 1-port and two port networks is given in Supplementary Section 1.

**Figure 4 | Series, shunt and quality factor frequency variations representations on a 2D plot and simultaneous 3D Smith chart visualization for the new fabricated Peano (pink) inductor in the conductive state of VO_2_ versus previously reported best performing inductor (red) using VO_2_ in same technology^37^. a,** 2D series inductance representation versus frequency: both Peano inductor and previously reported inductor^37^ show stable series inductance in within the 4GHz-10GHz frequency range. **b,** 2D frequency dependency of quality factors in the 4 GHz-15 GHz frequency range. **c,** Shunt inductance frequency dependency shows linearity on a sharper frequency range (see Supplementary Section 3) for both inductors. **d,** 3D Smith chart representation of series inductance over the S_11_ parameters in the 4 GHz-10 GHz frequency range. **e,** 3D Smith chart representation of series inductance and quality factors 4GHz-10GHz over the series inductances 3D curves. **f,** 3D Smith chart representation of series inductance and quality factors 4GHz-15GHz. The Qs start descending to 0 close to 15 GHz (the cylinders radius becomes 0 when Q becomes negative) for both models while the S_11_ parameters crossed into the West hemisphere of the Smith chart (capacitive region). **g,** 3D Smith chart representation of shunt inductance 4GHz-15GHz. One may see that the shunt inductance becomes negative below 15GHz (entering the 3D Smith chart) .**h**, 3D Smith chart representation of shunt inductance and quality factors 4GHz-15GHz.**i**, Simultaneously 3D Smith chart representation of series, shunt inductance and quality factors (along both series and shunt inductance) in the 4GHz-15GHz range.

**Figure 5 | Fabricated VO_2_ reconfigurable Peano inductor geometry and performances. a,** Cross sectional view **b,** SEM photo of the inductors. **c,** Current distribution (5GHz) in the off state for an inductor with 2 switches. The current ignores the VO_2_ switches and goes through all the windings in its path from port 1 (input) to port 2 (output). **d,** VO_2_ switch fabricated photo: a gap in the contact metal of 0.6μm is left in order to contact the VO_2_ layer. In the insulating phase of VO_2_ the switch is in off state (acting like a lossy dielectric), in the conductive state of the VO_2_ the 600nm gap plays an important role to minimize the conductive losses since the VO_2_ has a limited 48,000S/m conductivity on the SiO_2_/Si substrate. **e,** Current distribution (5GHz) in the hypothetical on state of one switch while the other is in the off state. The current ignores the off state VO_2_ switch and follows a shorter path from input to output. **f,** Current distribution (5 GHz) in the on state for an inductor with 2 switches. All current distributions are within a range from 0.5 A/m (dark blue) to 5.2 A/m (intense red) using the same scale. **g**, Series inductance of the fabricated inductors with 2 switches, in on state and off state, series inductance of an inductor with one switch in on state. **h**, Quality factor of the inductor with 2 switches in off state and on state, and of an inductor with one switch (on state). **i**, Simultaneous 3D Smith chart representation for the 2 GHz-10 GHz frequency of the series inductance and quality factors for all 3 situations.

**Figure 6 | Fabricated VO_2_ reconfigurable Peano inductors with additional various switches lengths and their influence in the on state. a,** Top view of the inductor-whose cross section is depicted in **Fig. 5 a**. **b,** SEM photo of a 2.3 μm long switch. **c,** SEM photo of a 1.5 μm long switch. **d,** Extracted ( from the measured S parameters): Lseries of the inductor with 2 switches in on state for 600 nm long switches, 1.5 μm and 2.3 μm. **e,** Q of the inductor with 2 switches in on state, for the 600 nm long switches, 1.5 μm and 2.3 μm. **f,** Simultaneous 3D Smith chart representation for the 4 GHz-10 GHz frequency of the Lseries, Q and S_11_ parameters for the three different inductors with 600 nm long switches, 1.5 μm and 2.3 μm long switches.

**Figure 7 | Proposed equivalent circuit of the Peano reconfigurable inductors and VO_2_ switches in the off and on state for the 4 GHz-10 GHz frequency range.** **a,** Off state modelling of the VO_2_ switch: R_VO2_off_ is modelling the undesired losses in between the adjacent turns where the VO_2_ switches are present. Ideally R_VO2_off_=∞, however the non-zero conductivity of VO_2_ (whose measurement in DC is present in Fig 9-Methods) will generate undesired conductive losses between turns. Similarly, the frequency dependent dielectric constant of the VO_2_ can in theory generate undesired couplings between the adjacent turns where the VO_2_ is present: C_VO2_off_. C_VO2_ground1_ and C_VO2_ground2_ are modelling the coupling losses between the VO_2_ switches and coplanar waveguide ground planes. **b,** Extracted equivalent circuit of the inductor: L_s_ stands for the series inductance, C_s_ stands for the inter-turns capacitance, C_ox1,_ C_ox2,_ C_sub1,_ C_sub2_, R_sub1,_ R_sub2_ describe the RF losses within the various substrates layers. R_p_ is modelling the undesired conductive losses between the adjacent turns due to the VO_2_ conductivity. In the off state we would like R_p_ to be ∞, however the conductive losses in the VO_2_ switches will make this term being un-unneglectable. R_S_ is modelling the series resistance-dependent on the Al deposition and trace width, (counting the on/off resistances corresponding to the current paths through the turns R_turns_on_/ R_turns_off_), but including too the VO_2_ resistive losses R_VO2_on_ in the on state of the switches. **c,** On state modelling of the VO_2_ switch: R_VO2_on_ is modelling the undesired resistive losses due to the limited conductivity of the VO_2_ in the on state. This resistance will contribute to a higher value of the R_s_ in the on state since the current will pass through the switches.

**Figure 8 | Fabricated VO_2_ reconfigurable Peano inductor equivalent circuit modelling within 4 GHz-10 GHz for the inductor with 2 switches, in on state and off state compared to measured ones. a** The resulted fitting of the equivalent circuit extracted Lseries_circuit and Lseries from the measured S parameters. The values are extracted optimizing simultaneously the Lseries_circuit, Q_circuit, module and phase of the S_ij_circuit_(jω) (2 scalar parameters and 3 complex values due to reciprocity). Off state values: L_S_=1.48 nH R_S_= 2.79 Ohms, R_p_=1341Ohms, C_s_=0.17fF, C_ox1_=C_ox2_=721 fF_,_ C_sub1_=C_sub2_=201 fF, R_sub1_=R_sub2_>10^8^Ohm. On state values: L_S_=0.90 nH, R_S_= 4.01 Ohms, R_p_=10^7^Ohm, C_s_=0.15fF, C_ox1_=C_ox2_=724 fF_,_ C_sub1_=C_sub2_=201 fF, R_sub1_=R_sub2_>10^8^Ohm. **b,** Scalar Q and extracted Q_circuit resulted fitting **c,** Simultaneous 3D Smith chart representation for Lseries_circuit, Q_circuit, S_ij_circuit_(jω), and Lseries, Q, S parameters of the measured inductors. The fitting of the S_21_ parameters is almost perfect, the fitting of the S_11_ parameters (whose values are extremely small in magnitude) is not perfect-but it overlaps on a specific frequency range too. S_11_ is more sensitive to the frequency dependency of the elements neglected in the simplified equivalent circuit in Fig.7b. **d,** Simultaneous 3D Smith chart representation for all the S_11_ (S_ij_circuit_(jω) and measured S_11_(jω)) parameters and their frequency dependency. It is interesting to notice their change of hemisphere just below 10 GHz for the off state modeled and measured inductors (this happening before their Q becomes 0-the radius of the cylinders in **c** is not yet zero, or in **b** one may see still the positive values of Q). This means that the imaginary part of S_11_ becomes “capacitive” before its Y_11_ imaginary part becomes 0-which determines the sign changes in Q. Referring to^56-57^ the classical quality factor^34-39^ analysed and implemented here too is a measure of the inductor performances while short-cutting the second port. If the inductor is connected in shunt in the final circuit the change of hemisphere of S_11_ below 10 GHz can be neglected, else depending on the configuration in which this is finally used- the change of hemisphere warns that under 50 Ohm load the inductor starts behaving capacitive before 10 GHz.

**Figure 9 – methods | Fabrication flow and VO_2_ characterization description. a,** VO_2_ deposition via PLD, followed by a photolithography step and dry etching of the VO_2_. **b,** Cr-20 nm/Al 400 nm contact metal deposition by evaporation after a subsequent photolithography and patterning on the VO_2_ **c**, Al evaporation and lift off for top metallization. **d,** top view of **a**. **e,** top view of **b**. **f, Top** view of **c**. **g,** grain size of the PLD VO_2_ deposition. **h,**  Conductivity levels in heating (red) and in cooling (blue). **i**, Switch SEM photo: The gap in the contact metal is below 600 nm over the entire width of the switches. This gap where only VO_2_ is active represents the switch length.

**Acknowledgments**

This work was supported by the HORIZON2020 FETOPEN PHASE-CHANGE SWITCH Project under Grant 737109. The work of E. Sanabria-Codesal was supported in part under DGCYT Grant MTM2015-64013-P.

**Author contributions**

A.A.M. proposed the new quantification model of the orientation of frequency over the 3D Smith chart, use of oriented curvature in the mathematical modelling, 3D series and shunt inductor Q inductor modelling, designed, fabricated and measured the devices with VO_2_. A.M. supervised and guided the overall 3D Smith chart tool new capabilities implementations for this article. V.A. contributed to the development of the new 3D Smith chart concepts, their actual Java implementation and revised and arranged the paper. E.S-C refined and contributed extensively to the oriented curvature mathematical computations (equations). R.A.K contributed with the fabrication and process flow in different stages, especially in obtaining the sub-micronic gaps in the Al via photolithography. A.K. contributed with the conductivity measurements of the VO_2_. M.F-B contributed with constructive critical view in the preliminary design stages. M.C made the PLD deposition of VO_2_. J.Z and E.C. contributed with the calibration of the measurement tool. A.S. supervised the work of A.K. A.M.I. lead the work of A.A.M and guided the implementation stages for each part of the work.

**Competing interests**

The authors declare no conflict of interest.

**Materials & Correspondence**

Correspondence and requests for materials should be addressed to A.A.M.
