# Supplementary material for: 3D Smith charts scattering parameters frequency-dependent orientation analysis and complex-scalar multi-parameter characterization applied to Peano reconfigurable vanadium dioxide inductors
Source: Sci Rep. 2019 Dec 4;9:18346. doi: 10.1038/s41598-019-54600-5 (PMC6892935; doi:10.1038/s41598-019-54600-5)
Supplement: Supplementary file 3 — Supplementary file [file 41598_2019_54600_MOESM3_ESM.docx]

**3D Smith charts scattering parameters frequency-dependent orientation analysis and complex-scalar multi-parameter characterization applied to Peano reconfigurable vanadium dioxide inductors**

Andrei A. Muller^1*^, Alin Moldoveanu^2^, Victor Asavei^2^, Riyaz A. Khadar^3^, Esther Sanabria-Codesal^4^, Anna Krammer^5^, Montserrat Fernandez-Bolaños^1^, Matteo Cavalleri^1^, Junrui Zhang^1^, Emanuele Casu^1^, Andreas Schuler^5^, Adrian M. Ionescu^1^

^1^ Nanoelectronic Devices Laboratory (NanoLab), École Polytechnique Fédérale de Lausanne (EPFL), 1015, Lausanne, Switzerland,

^2^ Department of Computer Science and Engineering, Faculty of Automatic Control and Computers,

University Politehnica of Bucharest, 060042 Bucharest, Romania,

^3^ Powerlab, École Polytechnique Fédérale de Lausanne (EPFL), 1015, Lausanne, Switzerland,

^4^ Departamento de Matemática Aplicada, Universitat Politècnica de València ,46022 Valencia, Spain,

^5^ Solar Energy and Building Physics Laboratory (LESO-PB), École Polytechnique Fédérale de Lausanne (EPFL), 1015, Lausanne, Switzerland.

^*^Correspondence to: [andrei.muller@epfl.ch](mailto:andrei.muller@epfl.ch)

1. **On the clockwise and anticlockwise frequency dependency of Foster and non-Foster circuits on the Smith chart**
   1. **Misleading remarks on the Counter-clockwise movement on the Smith chart**

In^1^ (year 2012) the authors state “The locus of the deembedded measured results rotates anti-clockwise on the Smith chart between 595MHz and 1.5GHz and this implies the presence of a non-Foster element”.

In^2^ (2012) “the result shows non-Foster behavior from 595MHz to 1.5GHz in the anticlockwise rotation of the traces”.

In^3^ (2013) “This exhibits the expected non-Foster performance by its anticlockwise rotation around the Smith chart with increasing frequency between 0.595 GHz and 1.5 GHz”.

In^4^ (2010) “It can be seen that both prototypes clearly show non-Foster behavior (locus of input impedance rotates counter-clockwise with frequency) within a very broad band (more than two octaves).

In^5^ (1957) “The admittance function plotted by the author on the Smith chart winds counterclockwise. This is contrary to the laws of nature for passive linear networks”. These laws prescribed that the plot of the admittance function wind clockwise from the low to the high frequency”.

- 1. **Counter-clockwise movements network containing Foster elements**

In^6-7^ (^6^Appendix B)-(^7^Appendix A) the so often misleading interpretation of counter-clockwise movement on the Smith chart as a direct consequence of a non-Foster element was noticed by B. A. Munk (1929-2009) where he gives empirically examples when this happens also for Foster elements (on a limited bandwidth).

- 1. **Correct statements on the Counter-clockwise movements network containing lossy non-Foster elements**

In^8^ it is stated (for the particular case of an antenna) “Usually, the impedance of any antenna moves about the Smith chart in a clockwise direction with increasing frequency. However, this behavior is not dependent on the antenna obeying the Foster reactance theorem”.

- 1. **Oriented curvature of a one port network**

We will construct our proposed theory based on the notion of oriented curvature^9-10^. Given a regular plane curve *C(s)=(r(s), x(s))*, i.e. *C'(s) ≠0*, we will call tangent vector *t(s) = C′(s)*. We consider that *C(s)* it is parametrized by the arc length, i.e*. <t(s), t(s)>=1*. In this case, the tangent vector can be completed with a vector *n(s)=(-x′(s), r′(s))*, so that {*t(s), n(s)*} form a positively oriented orthonormal base (the determinant of both vectors is positive). By using that *<t(s), t(s)>=1*, we obtain *2 <t′(s), t(s)>=0*, then *t′(s)* and *n(s)* have the same direction. We will call the derivative of the tangent vector *C''(s)=t′ (s)* the curvature vector of the curve. The oriented curvature *k(s)* is the projection of this curvature vector on the normal vector, thus

*k(s)= <n(s), C′′($s$)>=*$-x^{'}(s)r^{''}(s)+r^{'}\left( s \right)x^{''}(s)$ $= \left| \begin{matrix} r'(s) & x'(s) \\ r''(s) & x''(s) \end{matrix} \right|$ (S1)

The formula for the oriented curvature for an arbitrary parameterization of *C(ω)= r(ω)+j x(ω)* (where *ω* represents angular frequency) is obtained^13^ by using chain rule^10^:

*k(ω)*= $\frac{-x^{'}(\omega)r^{''}(\omega)+r^{'}\left( \omega\right)x^{''}(\omega)}{{({r^{'}(\omega)}^{2}+{x^{'}(\omega)}^{2})}^{3/2}}= \frac{\left| \begin{matrix} r'(\omega) & x'(\omega) \\ r''(\omega) & x''(\omega) \end{matrix} \right|}{{({r^{'}(\omega)}^{2}+{x^{'}(\omega)}^{2})}^{3/2}}$ (S2)

Then, the curvature sign is positive if the projection of *C′′($\omega$)* on the normal vector has the same direction as *n(ω)* and negative in other case. As *C′′($\omega$)* is the variation of the tangent vector *t($\omega$)* and the normal vector *n(ω)* completes the positively oriented orthonormal base {*t($\omega$)*, *n(ω)*} (i.e. it is always to the left of the tangent vector), then one gets that *k($\omega$)* is positive when the curve is turning "left" with respect to the tangent vector and negative when it turns "right”. Therefore, the oriented curvature of a plane curve gives us information about the orientation of the curve, and it is easy to see that the lines have zero curvature and that the curves with negative curvature are curves clockwise oriented and the curves with positive curvature are counterclockwise oriented. Consider first a one port network ended on a resistive load *r*. The input impedance $z_{m}\left( j\omega\right)$ is given by (S3), where $r_{m}\left( \omega\right)$ denotes its resistive part and $x_{m}\left( \omega\right)$ its reactive part, while its reflection coefficient is given by (S4). Computing the oriented curvature values for both of them we get $k_{zm}\left( \omega\right)$ (the oriented curvature of the input impedance) and $k_{\Gamma_{1zm}}\left( \omega\right)$ (the oriented curvature of the reflection coefficient) as (S5) and (S6).


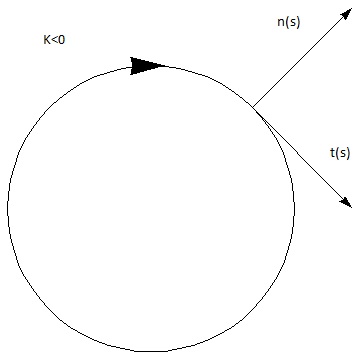

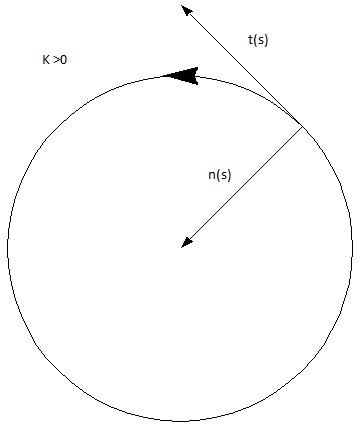
 **a** **b**


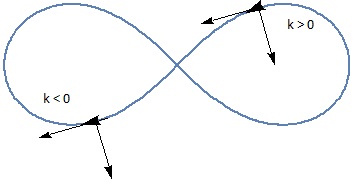
 **c**

**Supplementary Fig.1: Oriented curvature of a plane curve**. **a,**The oriented curvature *K* is positive. **b,** The oriented curvature K is negative **c**,The oriented curvature changes sign as one moves along it. Geometrically the circles in **a** and **b** are the same, from the differential geometry point of view they are different: having a different orientation dependent on their parametric equations. The geometrical image of reflection coefficients, input impedances may be the same for various circuits, however their motion on it will be determined by their differential geometry analysis of their curves-and thus the intrinsic difference between them can be detected.

$z_{m}\left( j\omega\right)=$ $r_{m}\left( \omega\right)+jx_{m}\left( \omega\right)$ (S3)

$\Gamma_{1zm}\left( j\omega\right)=\frac{z_{m}\left( j\omega\right)/r-1}{z_{m}\left( j\omega\right)/r+1}$ (S4)

$k_{zm}\left( \omega\right)$ =$\frac{-{x_{m}}^{'}\left( \omega\right){r_{m}}^{''}\left( \omega\right)+{r_{m}}^{'}\left( \omega\right){x_{zm}}^{''}\left( \omega\right)}{{({{r_{m}}^{'}\left( \omega\right)}^{2}+{{x_{m}}^{'}\left( \omega\right)}^{2})}^{3/2}}=\frac{{{r_{m}}^{'}\left( \omega\right)}^{2}\left( \frac{{x_{m}}^{'}\left( \omega\right)}{{r_{m}}^{'}\left( \omega\right)} \right)^{'}}{{({{r_{m}}^{'}\left( \omega\right)}^{2}+{{x_{m}}^{'}\left( \omega\right)}^{2})}^{3/2}}$ (S5)

$k_{\Gamma_{1zm}}\left( \omega\right)=\frac{{{{r_{m}}^{'}\left( \omega\right)}^{2}((r+r_{m}\left( \omega\right))}^{2}+{x_{m}\left( \omega\right)}^{2})\left( \frac{{x_{m}}^{'}\left( \omega\right)}{{r_{m}}^{'}\left( \omega\right)} \right)^{'}+2 {x_{m}\left( \omega\right)}^{2}({{r_{m}}^{'}\left( \omega\right)}^{2}+{{x_{m}}^{'}\left( \omega\right)}^{2})\left( \frac{r+r_{m}\left( \omega\right)}{x_{m}\left( \omega\right)} \right)^{'}}{2 r {({{r_{m}}^{'}\left( \omega\right)}^{2}+{{x_{m}}^{'}\left( \omega\right)}^{2})}^{3/2}}$ (S6)

Following (S 1)-(S 6) and **Supplementary Fig.1** one can easily conclude that^9-10^:

(i) If $k_{zm}\left( \omega\right)>0$ then $z_{m}\left( j\omega\right)$ is counter-clockwise oriented,

(ii) if $k_{zm}\left( \omega\right)<0$ then $z_{m}\left( j\omega\right)$ is clockwise oriented,

(iii) if $k_{\Gamma_{1zm}}\left( \omega\right)>0$ then $\Gamma_{1zm}\left( j\omega\right)$ is counter-clockwise oriented

(iv) $k_{\Gamma_{1zm}}\left( \omega\right)<0$ then $\Gamma_{1zm}\left( j\omega\right)$ is clockwise oriented.

(v) If $k_{zm}\left( \omega\right)=0$ then $z_{m}\left( j\omega\right)$ is a line.

(vi) If $k_{\Gamma_{1zm}}\left( \omega\right)=0$ then $\Gamma_{1zm}\left( j\omega\right)$ is a line.

In order that the orientation of the curves $z_{m}\left( j\omega\right)$ and $\Gamma_{1zm}\left( j\omega\right)$ should be the same, their curvatures must have the same sign. Paying attention to the formulas obtained for the curvature of $z_{m}\left( j\omega\right),$ the sign of $k_{zm}\left( \omega\right)$ depends only of $\left( \frac{{x_{m}}^{'}\left( \omega\right)}{{r_{m}}^{'}\left( \omega\right)} \right)^{'}$, because the rest of the factors are squared and therefore they are always positive, including r, which is a positive constant. However, to calculate the sign of $k_{\Gamma_{1zm}}\left( \omega\right),$ we will have to take into account $\left( \frac{{x_{m}}^{'}\left( \omega\right)}{{r_{m}}^{'}\left( \omega\right)} \right)^{'}$and $\left( \frac{r+r_{m}\left( \omega\right)}{x_{m}\left( \omega\right)} \right)^{'}$:

If $k_{zm}\left( \omega\right)>0 and\left( \frac{r+r_{m}\left( \omega\right)}{x_{m}\left( \omega\right)} \right)^{'}$> 0, we obtain $k_{\Gamma_{1Zm}}\left( \omega\right)>0$ (S7)

If $k_{zm}\left( \omega\right)<0 and\left( \frac{r+r_{m}\left( \omega\right)}{x_{m}\left( \omega\right)} \right)^{'}$< 0, we obtain $k_{\Gamma_{1Zm}}\left( \omega\right)<0$ (S8)

In any other case, the changes of sign between (S5) and (S6) as the angular frequency changes will depend on the sign of the numerator of the total expression (S6) and the following relation has to be obeyed so that both curvatures should have the same sign:

(S9)

This condition will assure that both curvatures have the same sign and therefore the same orientation.

- 1. **Prove that counter-clockwise frequency dependence can occur in linear passive circuits driving point immitance**

Any passive, causal, linear and stable network which can be realized by resistors, capacitors, inductors and transformers has a driving-port immitance characterized in the frequency domain by positive real functions^11-12^.

Let us consider a linear one port passive network represented by the following input impedance of the (S10). Making the notations in equations (S3)-(S4) for the real and imaginary part we obtain the expressions in equations (S10)-(S14). Considering the input port impedance *r=1*) we may compute the reflection coefficient with equations (S15)-(S17).

$z_{m}\left( s \right)=\frac{(s+4)(s+1)}{(s+2)(s+2.5)(s+1.5)}$ (S10)

$s=j\omega$ (S11)

$z_{m}\left( j\omega\right)=$ $r_{m}\left( \omega\right)+jx_{m}\left( \omega\right)$ (S12)

$r_{m}\left( \omega\right)=\frac{30+27.25\omega^{2}+\omega^{4}}{56.25+48.06\omega^{2}+12.5\omega^{4}+\omega^{6}}$ (S13)

$x_{m}\left( \omega\right)=\frac{-9.5\omega-14.25\omega^{3}-\omega^{5}}{56.25+48.06\omega^{2}+12.5\omega^{4}+\omega^{6}}$ (S14)

$\Gamma_{1zm}\left( j\omega\right)=\frac{z_{m}\left( j\omega\right)-1}{z_{m}\left( j\omega\right)+1}{=\Gamma}_{zmr}\left( \omega\right)+j\Gamma_{zmi}\left( \omega\right)$ (S15)

$\Gamma_{zmr}\left( \omega\right)=-\frac{40.25+31.0625\omega^{2}+11.5\omega^{4}+\omega^{6}}{132.25\text{ }+119.5625\omega^{2}+15.5\omega^{4}+\omega^{6}}$ (S16)

$\Gamma_{zmi}\left( \omega\right)=-\frac{19 \omega+28.5\omega^{3}-2\omega^{5})}{132.25\text{ }+119.5625\omega^{2}+15.5\omega^{4}+\omega^{6}}$ (S17)

Based on equations (S13) and (S14) the input impedance $z_{m}\left( j\omega\right)$ is plotted in **Supplementary Fig.2**. It has a clockwise orientation for -2< *ω<-0.19* and for 0.19< *ω<2*, while counter-clockwise orientation for -0.19< *ω<0.19*. Using (S16) and (S17) we present in **Supplementary Fig.2.b** the Smith chart plot of the reflection coefficient $\Gamma_{1zm}\left( j\omega\right)$. It has a clockwise orientation for -2< *ω<-0.28* and for 0.28< *ω<2*, while counter-clockwise orientation for -0.28< *ω<0.28*. Using (S5) and (S6) we plot the oriented curvatures of $z_{m}\left( j\omega\right)$ and $\Gamma_{1zm}\left( j\omega\right)$ in **Supplementary Fig.2. c**: $k_{zm}\left( \omega\right)<0$ for -2< *ω<-0.19* and for 0.19< *ω<2*, while $k_{zm}\left( \omega\right)>0$ for -0.19< *ω<0.19*. For $k_{\Gamma_{1zm}}\left( \omega\right)$ we get $k_{\Gamma_{1zm}}\left( \omega\right)<0$ for -2< *ω<-0.28* and for 0.28< *ω<2*, while $k_{\Gamma_{1zm}}\left( \omega\right)>0$ -0.28< *ω<0.28*. As described in (i) and (ii) the changes of sign in the curvature correspond to the changes of orientation in the $z_{m}\left( j\omega\right)$ and $\Gamma_{1zm}\left( j\omega\right)$. In **Supplementary Fig.2.** **d** one can see $x_{m}\left( \omega\right),$ it is a strictly monotonic decreasing function while sweeping angular frequency for -2<*ω<-2* since its derivative $\frac{dx_{m}\left( \omega\right)}{d\omega}<0$. It worth noting that the curvature changes in $z_{m}\left( j\omega\right)$ and $\Gamma_{1zm}\left( j\omega\right)$ occur for different values of *ω* and that counter-clockwise motion occurs for both of them on a limited frequency range.


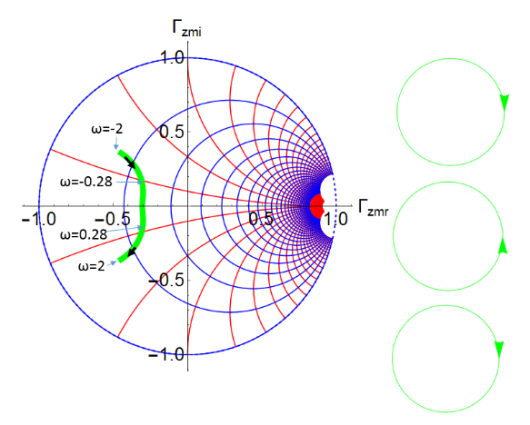

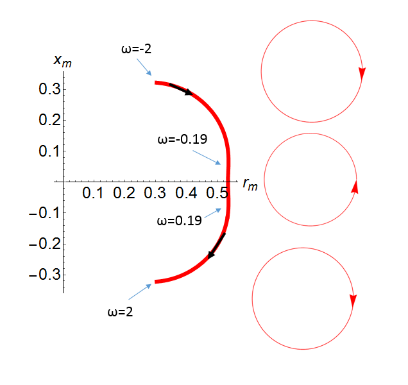
 **a b**


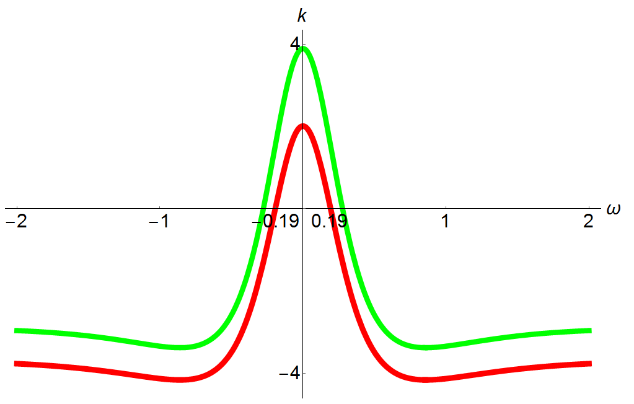
 **c d**


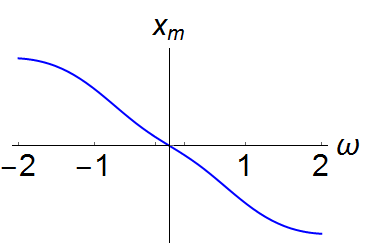


**d**

**Supplementary Fig. 2: Orientation and oriented curvature sign changes for the input impedance $\boldsymbol{z}_{\boldsymbol{m}}\left( \boldsymbol{j\omega} \right)$ in (S 10) and for its corresponding reflection coefficient $\boldsymbol{\Gamma}_{\boldsymbol{1}\boldsymbol{zm}}\left( \boldsymbol{j\omega} \right)\boldsymbol{.}$ a,** The input impedance $z_{m}\left( j\omega\right)$ (red), has mixed orientations, with orientation changes corresponding to the zeros of its oriented curvature $k_{zm}\left( \omega\right)$. **b,** The reflection coefficient $\Gamma_{1Zm}\left( j\omega\right)$ (green) has also mixed orientations on the Smith chart with the changes in it corresponding to the zeros of its oriented curvature $k_{\Gamma_{1Zm}}\left( \omega\right)$. **c,** The oriented curvatures for $z_{m}\left( j\omega\right)$ and $\Gamma_{1Zm}\left( j\omega\right)$: $k_{zm}\left( \omega\right)$ (red) and $k_{\Gamma_{1Zm}}\left( \omega\right)$ (green). They change sign at ω= ±0.19 and ω= ±0.28. **d**, The $x_{m}\left( \omega\right)$ has no monotony changes for -0.28< *ω<0.28*.

Circuits including non-Foster elements can surely exhibit too counter-clockwise Smith chart rotations (for their reflection coefficients or input impedances) on certain frequency ranges. The non-Foster elements, which do not obey the Foster reactance theorem^13-15^ (which is for purely reactive networks), given in (S18) (where *X* stands for reactance and *B* stands for the susceptance) play a major role in the bandwidth enhancement, being extensively used in small antenna designs. However, in practical realization they are always lossy^13^, (being not purely reactive). In **Supplementary Fig. 3**. we can see the S_11_ parameters of a network including Non-Foster elements (provided with the extremely kind amiability of Prof. R.W. Ziolkowski), employed in ^13^.

**a** **b** **c**


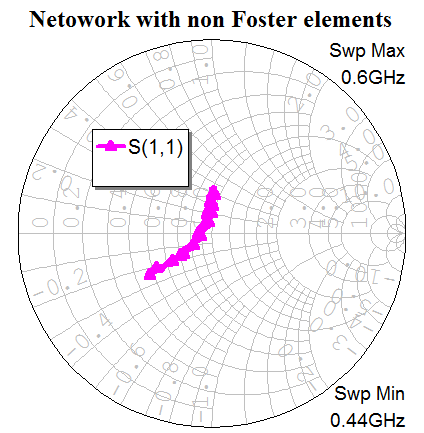


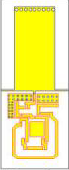

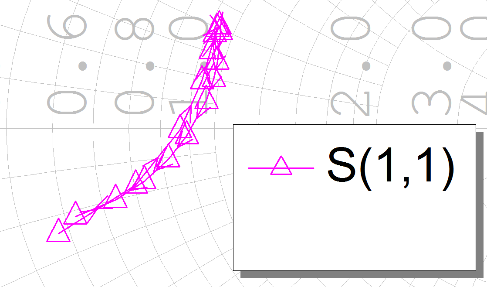


**Supplementary Fig. 3: Circuit with non-Foster elements^13^ a,** (S parameters provided with kindness by R.W. Ziolkowski). **b,** Clockwise and counter-clockwise movement of the S parameters occurs but may be overlooked and may be uneasy to be detected on a classical Smith chart even when zooming. Please look on **Supplementary** **Video**. The circuit changes orientation of the S_11_ parameter at 0.49 GHz. Circuit is analyzed between 0.44 GHz and 0.60 GHz. **c,** Circuit layout

- 1. **Foster circuits –purely clockwise movement**

Let us first consider a simple one port circuit (**Supplementary Fig. 4a)** composed just by ideal lossless inductors and capacitors: its driving port impedance reactance $x_{F}\left( \omega\right)$ is given by (S19) (a), where which is monotonically increasing with the angular frequency *ω* - Foster theorem^7-8,12,14-15^ (S18). Its 1-port $\Gamma_{1F}\left( j\omega\right)$ reflection coefficient is given in (S19) (b). Considering now the same Foster network in a two port (**Supplementary Fig. 4b)** connection (assuming identical ports impedances *r*) one can compute the reflection coefficient as $\Gamma_{2F}\left( j\omega\right)$ (S19) (c). Calculating now the associated oriented curvatures of (S19) (a)-(c), we obtain $k_{z}\left( \omega\right)$*,* $k_{\Gamma1F}\left( \omega\right), k_{\Gamma2F}\left( \omega\right)$, i. e. the expressions (S20) (a), (b) and (c). The oriented curvature of the one port Foster network $k_{\Gamma1F}\left( \omega\right)$= -1 implies that the reflection coefficient of the 1-port Foster networks will have a clockwise orientation as $\omega$ increases (see statement (ii)). The oriented curvature the $k_{\Gamma2F}\left( \omega\right)$= -2 implies the same (since its sign is negative).


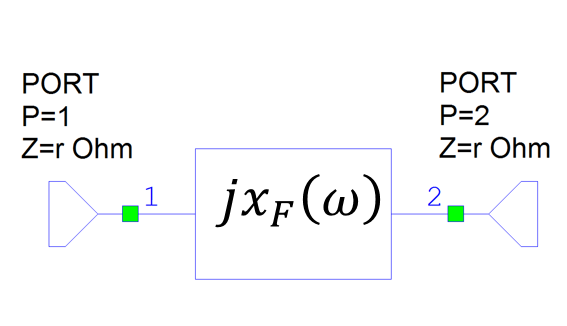
 **a b**


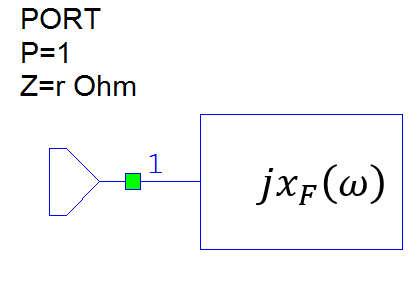


**Supplementary Fig. 4: One port and two port networks with Foster circuit elements $\boldsymbol{.}$ a,** One port circuit containing only Foster elements $z_{m}\left( j\omega\right)={jx}_{F}\left( \omega\right)$ **b,** Two port circuit containing only Foster elements.

$\frac{dx_{F}\left( \omega\right)}{d\omega}>0 and \frac{dB_{F}\left( \omega\right)}{d\omega}>0$ (S18)

(a) $z_{F}\left( j\omega\right)=jx_{F}\left( \omega\right)$ (b) $\Gamma_{1F}\left( j\omega\right)=\frac{jx_{F}\left( \omega\right)/r-1}{jx_{F}\left( \omega\right)/r+1}$ (c) $\Gamma_{2F}\left( j\omega\right)=\frac{jx_{F}\left( \omega\right)}{jx_{F}\left( \omega\right)+2}$ (S19)

(a) $k_{Fz}\left( \omega\right)$= 0, (b) $k_{\Gamma1F}\left( \omega\right)$= $-\frac{r{x_{F}}^{'}\left( \omega\right)}{\left( r^{2}+{x_{F}\left( \omega\right)}^{2} \right)\sqrt{\frac{r^{2}{{x_{F}}^{'}\left( \omega\right)}^{2}}{\left( r^{2}+{x_{F}\left( \omega\right)}^{2} \right)^{2}}}}=-1,$ (c) $k_{\Gamma2F}\left( \omega\right)$= = $-\frac{2 (4+{x_{F}\left( \omega\right)}^{2})\sqrt{\frac{{{x_{F}}^{'}\left( \omega\right)}^{2}}{\left( 4+{x_{F}\left( \omega\right)}^{2} \right)^{2}}}}{{x_{F}}^{'}\left( \omega\right)}=-2$ (S20)

The reflection coefficients $\Gamma_{1F}\left( j\omega\right)$ and $\Gamma_{2F}\left( j\omega\right)$ are direct inversive transformation of $z_{F}\left( j\omega\right)$ (simple Möbius transformation)^16,17^ having the form (S21) (where *a*, *b*, *c*, *d* are constants and $f(\omega$) any real valued function)

$\Gamma\left( j\omega\right)=\frac{a*jf\left( \omega\right)+b}{c*jf\left( \omega\right)+d}$ (S21)

and thus^17^ they map the oriented line of $jx_{F}\left( \omega\right)$ (imaginary axes of the impedance plane) into circles with clockwise orientation, i. e. the curvature $k_{\Gamma1F}\left( \omega\right)<0$ and $k_{\Gamma2F}\left( \omega\right)<0$. Additionally, $1/{|k}_{\Gamma1F}\left( \omega\right)|=1,$ $1/{|k}_{\Gamma2F}\left( \omega\right)|=0.5,$ thus the reflection coefficients will move on the unit circle, respectively on a 0.5 radius circle.

- 1. **Non-Foster circuits –purely anti-clockwise movement**

Let us first consider a simple one port circuit (**Supplementary Fig. 5a**) composed just by ideal lossless inductors and capacitors but with negative inductances and capacitances^14-15^: the driving point reactance $x_{NF}\left( \omega\right)$(susceptance) obey (S22) while it can be always written as (S 23) where $x_{F1}\left( \omega\right)$ is a Foster reactance obeying (S18). Its 1-port $\Gamma_{1NF}\left( j\omega\right)$ reflection coefficient is given in (S 25). Considering now the same Non-Foster network in a two port (**Supplementary Fig. 5 b**) connection (assuming identical ports impedances *r*) one can compute the reflection coefficient as $\Gamma_{2NF}\left( j\omega\right)$ (S24) (c). Calculating now the associated oriented curvatures of (S24) (a)-(c) , i. e. $k_{Nz}\left( \omega\right)$*,* $k_{\Gamma1NF}\left( \omega\right), k_{\Gamma2NF}\left( \omega\right)$, we get (S25) (a), (b) and (c).The oriented curvature of the one port Foster network $k_{\Gamma1NF}\left( \omega\right)$= 1 implies (statement (iii)) that the reflection coefficient of the non-Foster 1 port networks will have a counter-clockwise orientation as $\omega$ increases. The oriented curvature the $k_{\Gamma2F}\left( \omega\right)$= 2 implies the same for the two port case scenario ( since its sign is positive).

**a b**


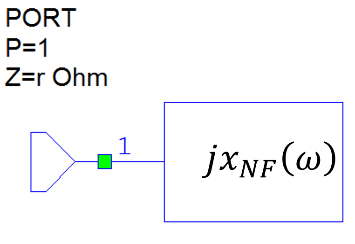

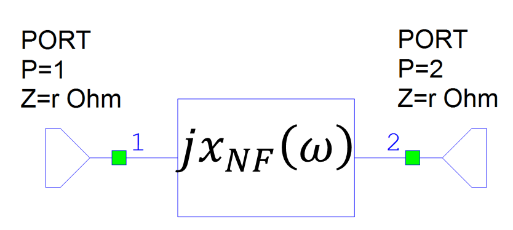


**Supplementary Fig. 5: One port and two port networks with purely non-Foster circuit elements^14-15^ $\boldsymbol{.}$ a,** One port circuit containing only Non-Foster elements $z_{m}\left( j\omega\right)={jx}_{NF}\left( \omega\right)$ **b,** Two port circuit containing only Non- Foster elements.

$\frac{dx_{NF}\left( \omega\right)}{d\omega}<0 and \frac{dB_{NF}\left( \omega\right)}{d\omega}<0$ (S22)

$x_{NF}\left( \omega\right)=-x_{F1}\left( \omega\right)$ (S23)

(a) $z_{NF}\left( j\omega\right)=-jx_{F1}\left( \omega\right)$ (b) $\Gamma_{1NF}\left( j\omega\right)=\frac{-jx_{F1}\left( \omega\right)/r-1}{-jx_{F1}\left( \omega\right)/r+1}$ (c) $\Gamma_{2NF}\left( j\omega\right)=\frac{-jx_{F}\left( \omega\right)}{-jx_{F}\left( \omega\right)+2}$ (S24)

(a) $k_{NFz}\left( \omega\right)$= 0, (b) $k_{\Gamma1NF}\left( \omega\right)$=$\frac{r{x_{F}}^{'}\left( \omega\right)}{\left( r^{2}+{x_{F}\left( \omega\right)}^{2} \right)\sqrt{\frac{r^{2}{{x_{F}}^{'}\left( \omega\right)}^{2}}{\left( r^{2}+{x_{F}\left( \omega\right)}^{2} \right)^{2}}}}=$ $1,$ (c) $k_{\Gamma2NF}\left( \omega\right)$=$\frac{2 (4+{x_{F}\left( \omega\right)}^{2})\sqrt{\frac{{{x_{F}}^{'}\left( \omega\right)}^{2}}{\left( 4+{x_{F}\left( \omega\right)}^{2} \right)^{2}}}}{{x_{F}}^{'}\left( \omega\right)}=$ $2$ (S25)

The reflection coefficients $\Gamma_{1NF}\left( j\omega\right)$ and $\Gamma_{2NF}\left( j\omega\right)$ are indirect inversive transformation of $jx_{F1}\left( \omega\right)$ (simple Möbius transformation)^16,17,18^ having the form (S 26) (where *a*, *b*, *c*, *d* are constants and $f(\omega$) any real valued function)

$\Gamma\left( j\omega\right)=\frac{a*conj(jx_{F1}\left( \omega\right))+b}{c*conj(jx_{F1}\left( \omega\right))+d}=\frac{-ajx_{F1}\left( \omega\right)+b}{-cjx_{F1}\left( \omega\right)+d}$ (S26)

and thus^17^ they map the oriented line of $jx_{F}\left( \omega\right)$ (imaginary axes of the impedance plane) into circles with counter-clockwise orientation, i. e. $k_{\Gamma1F}\left( \omega\right)>0$ and $k_{\Gamma2F}\left( \omega\right)>0$. Additionally, $1/{|k}_{\Gamma1F}\left( \omega\right)|=1,$ $1/{|k}_{\Gamma2F}\left( \omega\right)|=0.5,$ thus the reflection coefficients will move on the unit circle, respectively on a 0.5 radius circle.

1. **New frequency orientation quantification, series and shunt inductance representations and fabricated inductor frequency dependent quality factors implementations over the 3D Smith chart tool**
   1. **3D Smith chart concept previous capabilities**

The 3D Smith chart tool was released in 2017 and is based on the articles^18-22^. The 3D Smith chart tool generalizes the Smith chart^23^. The Smith chart became throughout the years a tool widely used in engineering in different areas for displaying of reflection coefficients^24-31^ being extensively used in the design stage or measurement phase of a variety of circuits in the RF, microwaves and THz frequency range.

The Smith chart main equation is given in (S27) and is applied to the grid of normalized impedance plane resulting in **Supplementary Fig. 6 a**. Equation (S27) maps the right half plane of the impedance plane into the unit circle of the reflection coefficients plane. The left half plane is mapped in its exterior **Supplementary Fig. 6 b** which extends to infinity. For this reason, circuits exhibiting negative part of the input impedance cannot be analyzed on the Smith chart, while with difficulty on the extended Smith chart.

$\Gamma\left( z \right)=\frac{z-1}{z+1}$ z∈{ℂ} (S27)

**a** **b**


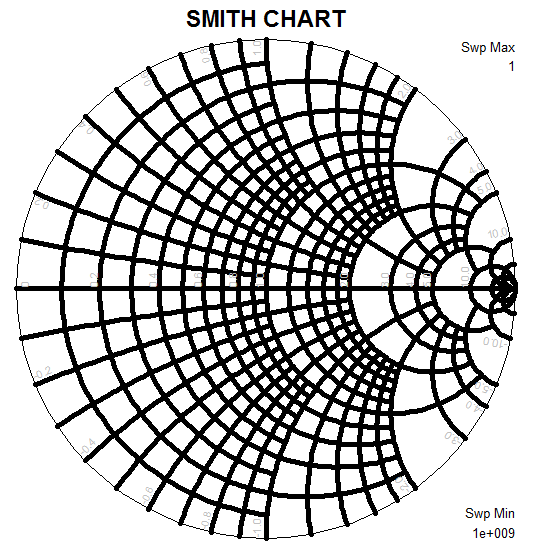

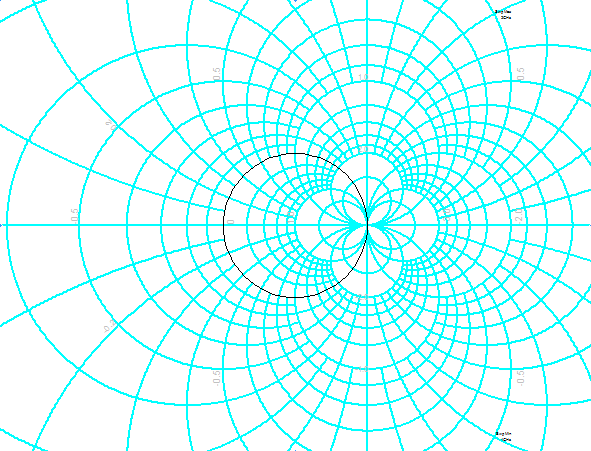


**Supplementary Fig. 6: Smith chart and extended 2D Smith chart $\boldsymbol{.}$ a,** The regions with reflection coefficient bigger than unity in absolute value cannot be seen **b,** The regions with reflection coefficient bigger than unity extend to infinity thus the chart cannot be easily used for circuits with negative part of the real part of the input impedance.

The 3D Smith chart main equation is also (S27) but it is considered that z∈ℂ⋃{∞} and using Riemann- Maxime Bochert representation of Mobius transformations^16-17^ ( in order to preserve circle shapes in a compact space) we get based on **Supplementary Fig. 7 a** and **b** in **Supplementary Fig. 8**.


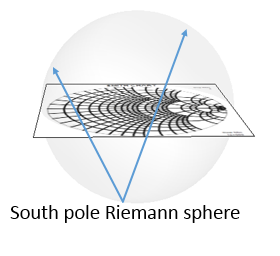
 **a b**


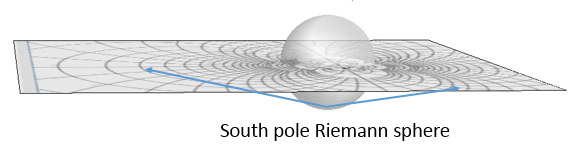


**Supplementary Fig. 7: Smith chart and extended 2D Smith chart mapping on the Riemann sphere$\boldsymbol{.}$ a,** The regions with reflection coefficients magnitude smaller than unity are mapped into the north hemisphere. **b,** The regions with reflection coefficient magnitudes bigger than unity are mapped into the South hemisphere.

**
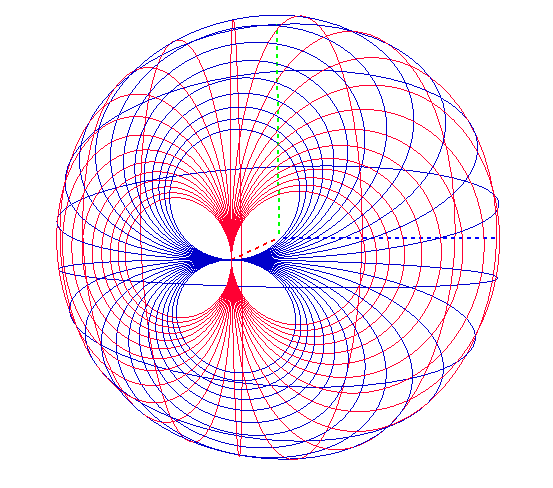
**

**Supplementary Fig. 8: 3D Smith chart:** South hemisphere: circuits with reflection coefficients magnitude bigger than unity. North hemisphere: circuits with reflection coefficients magnitude smaller than unity. East: Inductive, West: Capacitive.

- 1. **Implementations details for the 3D Smith Chart Application**

The 3D Smith Chart application is developed using the Java programming language and the following libraries are used:

- For the 3D rendering the OpenGL API is being used through the Java Binding for the Open GL API (JOGL2) library with multi-platform support on 32/64 bits;
- The Apache Commons Mathematics Library is being used for mathematical computations and program data structures (representation of complex numbers, fast matrix multiplication representation);

The NetBeans IDE with Beans Binding Library is used to build the GUI of the application.

- - 1. **Frequency visualization**

A new mode of visualization of the frequency associated to each point of the 3D space curve (computed using the $S_{11}$ (complex) reflection parameter) has been developed for the present paper. Each frequency that corresponds to a point of the 3D curve will be displayed as a segment on the line that passes from the center of the 3D sphere and the point of the 3D curve. The length of the segment will be given by the normalized frequency and the direction will be outwards of the surface of the 3D sphere.

To visualize the frequency dependency, the following steps are done:

For each point $P$ of the 3D curve:

Compute the direction vector defined by the center of the sphere and the point of the 3D curve

$dir\left( X,Y,Z \right)=\left( P\left( X \right)-sphereCenter\left( X \right),P\left( Y \right)-sphereCenter\left( Y \right),P\left( Z \right)-sphereCenter\left( Z \right) \right)$

Normalize the direction vector

${dir}_{N}(X,Y,Z)=\frac{dir(X,Y,Z)}{\left| dir(X,Y,Z) \right|}$ (S28)

Normalize the frequency associated to each point of the 3D curve

$f_{N}=\frac{f}{Max \left( f \right)}$ (S29)

Compute $P_{END}$

$P_{END}(X,Y,Z)= \left( {dir}_{N}\left( X \right)*f_{N}, {dir}_{N}\left( Y \right)*f_{N},{dir}_{N}\left( Z \right)*f_{N} \right)$ (S30)

Draw the segment [$P_{START}P_{END}$] where $P_{START}$ is P, the point of the 3D curve.


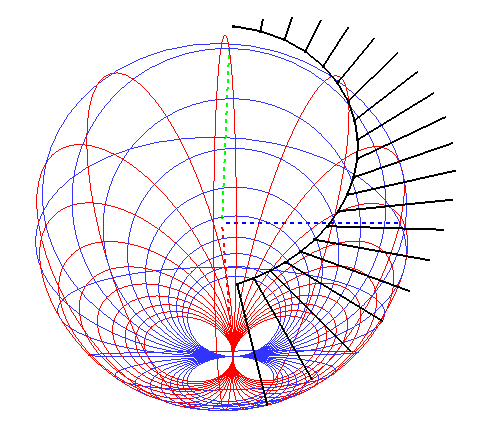


**Supplementary Fig. 9**: Frequency displayed as segment on a LSeries 3D curve.

**Note**: if multiple circuits (multiple touchstone files) are represented then $Max \left( f \right)$, used in the normalization of the frequency to obtain $f_{N}$, is the one greater across all the circuits used.

- - 1. **New parameters implemented**

For the present paper, the following new parameters have been implemented in the 3D Smith Chart application:

- Lseries
- LShunt

QualityFactor for Lseries/LShunt

**Lseries**

Lseries is computed using the following formula:

$Lseries\left( \omega\right)=Imag\left( \frac{R(1+ S_{11}- S_{12}S_{21}+ S_{22}+ S_{11}S_{22} )}{2*Pi*f*2* S_{21}} \right)$ (S31)

Then $LSeries\left( \omega\right)$ is normalized

${Lseries}_{N}\left( \omega\right)=\frac{LSeries\left( \omega\right)}{Max \left( LSeries\left( \omega\right) \right)}$ (S32)

To obtain the 3D representation of Lseries the following transformation is done

${Lseries}_{3d}\left( \omega\right)= \left( {LSeries}_{N}\left( \omega\right)+1 \right)* S_{{11}_{3d}}\left( j\omega\right)$ (S33)

Where $S_{{11}_{3d}}\left( j\omega\right)$ is^21^

$S_{{11}_{3d}}\left( j\omega\right)=Imag\left( \frac{2a(\omega)}{1+\left| S_{11}(\omega) \right|^{2}} \right.,\frac{2b(\omega)}{1+\left| S_{11}(\omega) \right|^{2}},\left. \frac{1-\left| S_{11}(\omega) \right|^{2}}{1+\left| S_{11}(\omega) \right|^{2}} \right)$ (S34)

**
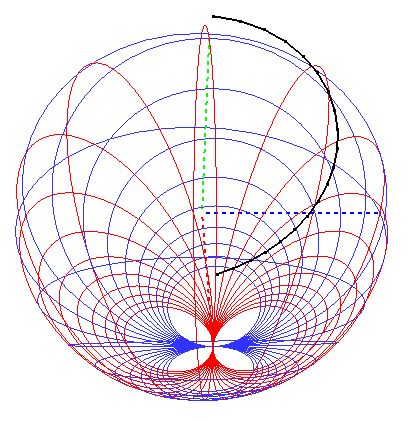
**

**Supplementary Fig. 10**: Lseries 3D curve (black) displayed above the 3D Smith chart unit ball. Please note that unlike the S parameters which are on the unit sphere, the normalized inductance in the 3D space.

**LShunt**

LShunt is computed using the following formula:

$LShunt\left( \omega\right)=Imag\left( \frac{1}{y_{11}} \right)* \frac{1}{2*PI*f}$ (S35)

Where $y_{11}$ is

$y_{11}=\left( \frac{1+S_{12}S_{21}+S_{22}- S_{11}(1+S_{22} )}{R(1+ S_{11}- S_{12}S_{21}+ S_{22}+ S_{11}S_{22} )} \right)$ (S36)

Then $LShunt\left( \omega\right)$ is normalized

${LShunt}_{N}\left( \omega\right)=\frac{LShunt\left( \omega\right)}{Max \left( LShunt\left( \omega\right) \right)}$ (S37)

To obtain the 3D representation of LShunt the following transformation is done

${LShunt}_{3d}\left( \omega\right)= \left( {LShunt}_{N}\left( \omega\right)+1 \right)* S_{{11}_{3d}}\left( j\omega\right)$ (S38)

Where $S_{{11}_{3d}}\left( j\omega\right)$ is defined in (S 34).

**Note**: if multiple circuits (multiple touchstone files) are represented then the $Max \left( LSeries\left( \omega\right) \right)$ / $Max \left( LShunt\left( \omega\right) \right)$, used in the normalization step to obtain ${LSeries}_{N}$ / ${LShunt}_{N}$, is the one greater across all the circuits used.

**QualityFactor for Lseries/LShunt**

The QualityFactor is computed using the following formula:

$Q\left( \omega\right)= -\frac{Imag\left( y_{11} \right)}{Real\left( y_{11} \right)}$ (S39)

The $Q\left( \omega\right)$ is normalized

$Q_{N}\left( \omega\right)=\frac{Q\left( \omega\right)}{Max \left( Q\left( \omega\right) \right)}$ (S40)

**Note**: if multiple circuits (multiple touchstone files) are represented then the $Max \left( Q\left( \omega\right) \right)$, used in the normalization step to obtain $Q_{N}\left( \omega\right)$, is the one greater across all the circuits used.

Using the 3D representation of the Lseries / Lshunt curve we can use the normal plane of the curve to associate to each point of the curve its quality factor as a cylinder of variable radius. This process will be described in the next section.

- - 1. **3D representation of the QualityFactor** $Q_{3D}\left( \omega\right)$

For the 3D representation of the Lseries / Lshunt, the normalized Lseries / Lshunt value and the $S_{11}$transmission parameter are used and thus each point of the 3D curve is computed using the following formula:

$L_{3d}\left( \omega\right)= \left( L_{N}\left( \omega\right)+1 \right)* S_{{11}_{3d}}\left( j\omega\right)$ (S41)

For the 3D representation that uses the quality factor as a 3D variable radius cylinder, the following steps have been implemented for each segment of the 3D space curve:

1. **Draw the 3D cylinder**

For each segment [$P_{i}P_{i+1}$] of the Lseries / Lshunt 3D space curve, a cylinder is drawn along the Z axis with the base of the cylinder centered in $P_{i}$ and the top of the cylinder centered in $P_{i+1}$. The radiuses of the base and of the top of the cylinder will have the values associated to the normalized quality factors $Q_{N}\left( \omega\right)$ for point $P_{i}$ and $P_{i+1}$ respectively. The height of the cylinder will have the value of the length of the [$P_{i}P_{i+1}$] segment of the curve.


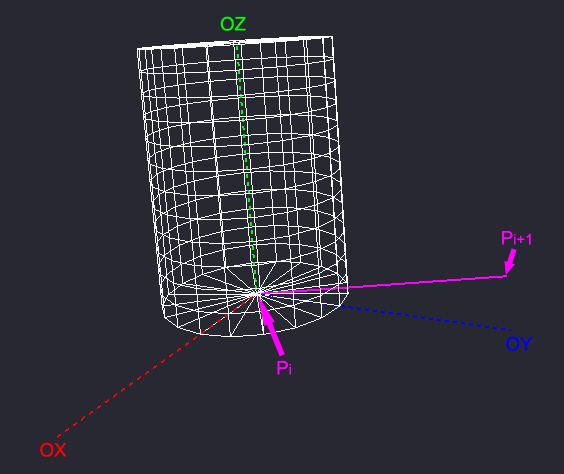


**Supplementary Fig. 11**: 3D cylinder for the [$P_{i}P_{i+1}$] segment of the curve drawn along the Z axis.

1. **Compute transformation parameters for the 3D cylinder**

To align the 3D cylinder along the [$P_{i}P_{i+1}$] segment the following parameters are computed:

- the angle between the OZ axis and the [$P_{i}P_{i+1}$] segment as the dot product
- the axis around which the 3D cylinder will be rotated as the cross product between the OZ axis and the [$P_{i}P_{i+1}$] segment


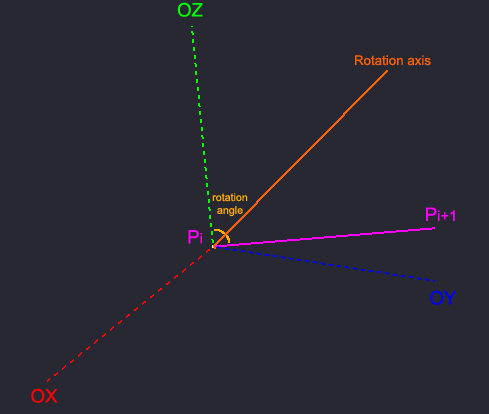


**Supplementary Fig. 12**: Transformation parameters (rotation angle and rotation axis) for the 3D cylinder.

1. **Apply the transformation to the 3D cylinder**

The 3D cylinder will be aligned along the [$P_{i}P_{i+1}$] segment ($P_{i}$ will be at the center of the base of the cylinder and $P_{i+1}$will be at the center of the top of the cylinder) by rotating the 3D cylinder around the rotation axis and using the angle computed in the previous step.


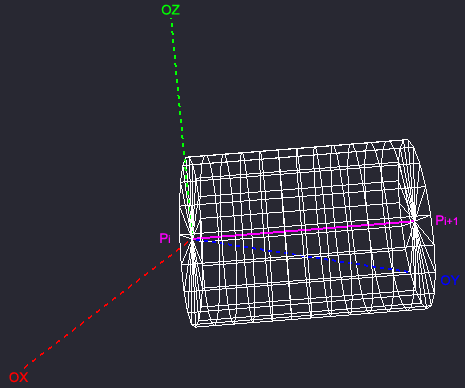


**Supplementary Fig. 13**: The $Q_{3D}\left( \omega\right)$ 3D cylinder transformed so that it wraps around the [$P_{i}P_{i+1}$] segment of the curve.

The steps described above will be applied to each segment of the 3D space curve and the result can be seen in the picture **Supplementary Fig. 14** below:


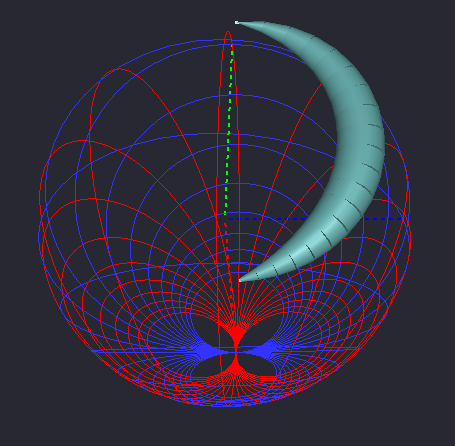


**Supplementary Fig. 14**: Final representation using 3D $Q_{3D}\left( \omega\right)$ cylinders in solid and lit mode.

1. **Inductances and quality factors of fabricated (reconfigurable) inductors on the 3D Smith chart**

It is important to stress that there are several models for extracting the inductance of a high frequency range inductor. For example the models in^34-35^ use the Y_21_ to extract its value (or equivalently this means using the parameter B of the ABCD matrix):

$\left[\begin{matrix} V_{1} \\ I_{1} \end{matrix}\right]=\left[\begin{matrix} A & B \\ C & D \end{matrix}\right]\bullet\left[\begin{matrix} V_{2} \\ -I_{2} \end{matrix}\right]$ (S42)

$\left[\begin{matrix} A & B \\ C & D \end{matrix}\right]=\left[\begin{matrix} 1 & j\omega L \\ 0 & 1 \end{matrix}\right]$ (S43)

Using the simplified PI model of an inductor shown in **Supplementary** Fig. 15 we may see that this model neglects in its implementation the shunt capacitance:

ωL_series_=$-Imag\left( \frac{1}{y_{21}} \right)=Imag(B)$=Imag( (R_s_+ jωL)||()) (S44)

**a b**


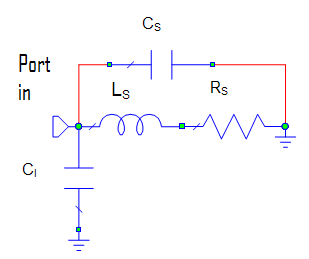

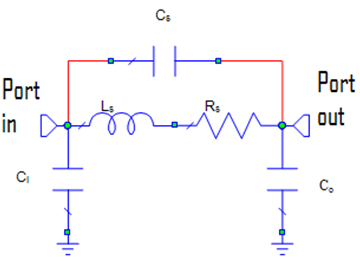


**Supplementary Fig. 15**: Simplified Pi model of an inductor. **a,** simplified Pi model **b**, Simplified Pi model with a port connected to the ground.

In^36-38^ authors use the Y_11_ (or equivalently parameter C) model for computing thus, in their model the second port is connected to the ground (**Supplementary Fig. 15 b**). For the simplified model in **Supplementary Fig. 15** this makes L_shunt_ be extracted from (S45)

ωL_shunt_=Imag((R_s_+jωL)||( )||($\frac{1}{j\omega C_{1}}$)) (S45)

Nevertheless both models can be alternatively used, since an inductor can be placed in between various circuits in various configurations^39^. Concerning the quality factor we adopt the common used model (S39)^34-38^. In **Supplementary** **Fig. 16** we can see the performances of the newly designed Peano inductor in respect to the previously reported inductor with VO_2_ in the same technology ( in the conductive, on state of VO_2_).

Considering the new designed inductors extracted inductances and quality factor for two different frequency ranges: consider **Supplementary Fig. 17 - Supplementary Fig. 21.** (the new Peano layout is presented in **Supplementary Fig.** 22)

**a b c**


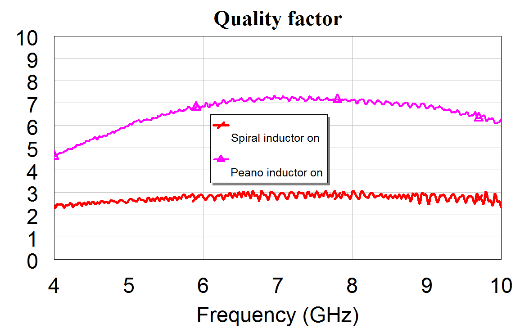

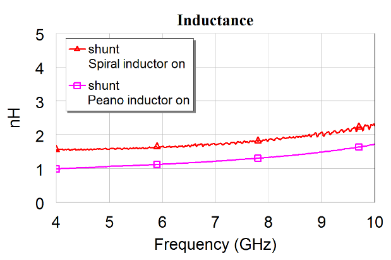

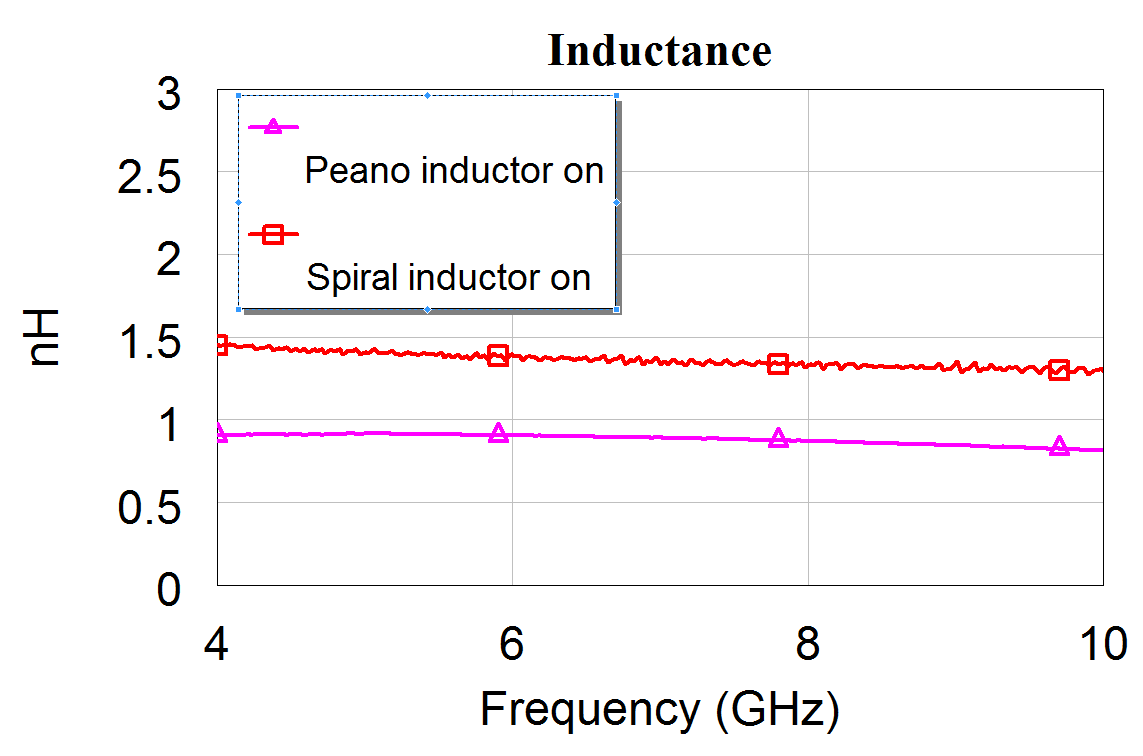


**Supplementary Fig. 16**: Comparative analyze of the new Peano inductor in the conductive state of VO_2_ versus previously reported reconfigurable inductor based on VO_2_ in the on state. (4GHz-10 GHz): a series model of inductance, b: shunt model, c: quality factor. Peano inductor (purple), previous^34^ reported inductor with VO_2_ –red.

**a b c d**


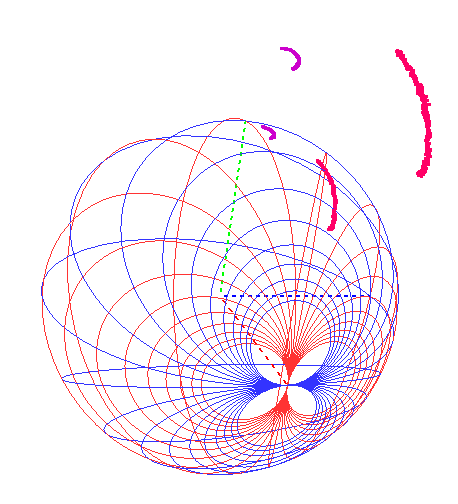

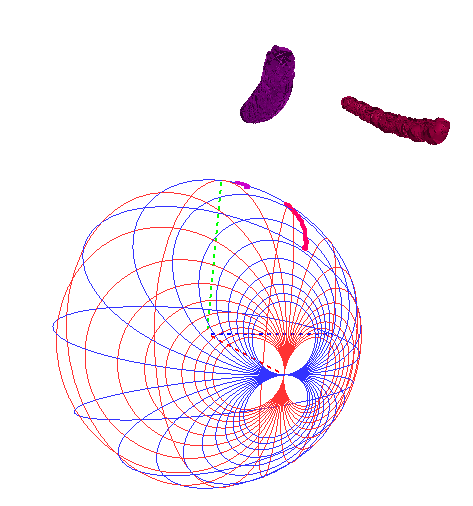

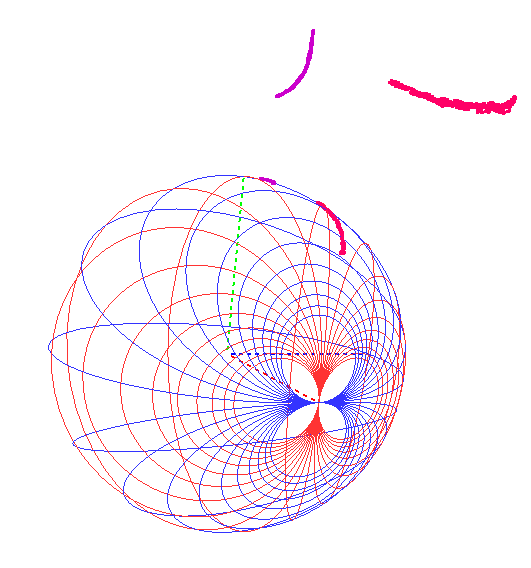


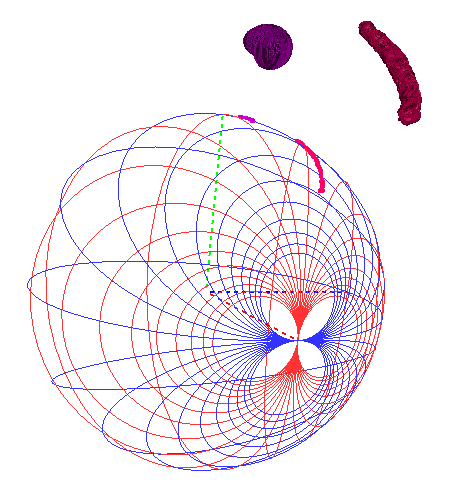


**Supplementary Fig. 17**: Comparative analyze of the new Peano inductor in the conductive state of VO_2_ versus previously reported^34^ reconfigurable inductor based on VO_2_ in the on state. (4GHz-10 GHz) on the 3D Smith chart (corresponding to Supplementary Fig. 21 layout): a series model of inductance, b: shunt model, c: quality factor. On the 3D smith chart one can see the S_11_ parameter to which they correspond. Please check video for better visualizations.

**
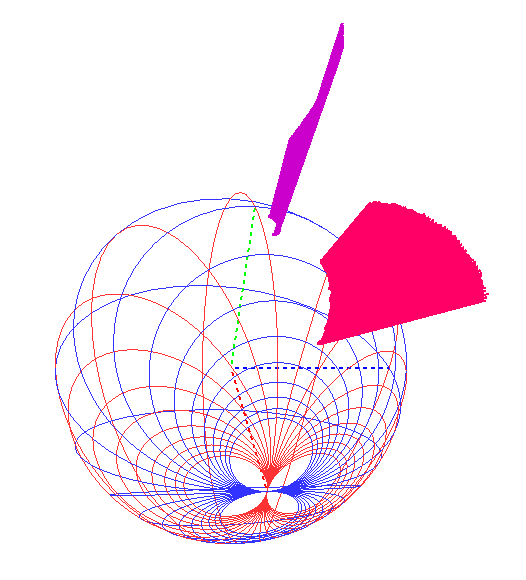
Supplementary Fig. 17a** contains more (indirect) information about the shunt inductance too, it shows that up to 10 GHz both inductors are still in the East hemisphere. In **Supplementary Fig. 17b** we can see how the shunt inductance model start being non-linear. In **Supplementary Fig. 17 c** and **d** we can see both quality factors plotted along the inductances, their frequency dependency being visible. In **Supplementary Fig. 18** we can see the clockwise orientation with increasing frequency of the S_11_ reflection parameter for both inductors.

**Supplementary Fig. 18**: Comparative analyze of the new Peano inductor in the conductive state of VO_2_ versus previously reported reconfigurable inductor based on VO_2_ in the on state. (4GHz-10 GHz): up to 10 GHz, both inductors stay in the inductive hemisphere (East), rotating clockwise as frequency increases.

**a b c**


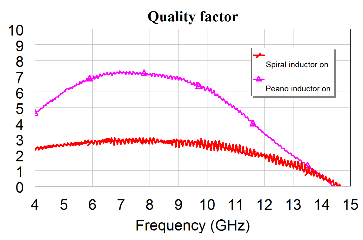

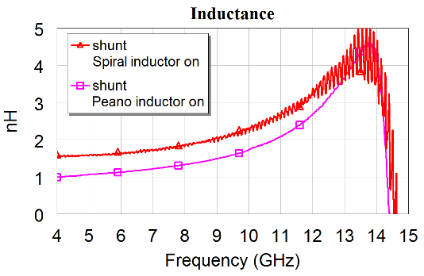

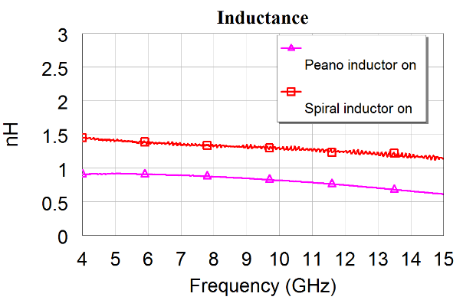


**Supplementary Fig. 19**: Comparative analyze of the new Peano inductor in the conductive state of VO_2_ versus previously reported reconfigurable inductor based on VO_2_ in the on state. (4GHz-15 GHz): **a**, series inductance, b: shunt inductance, c: quality factor.


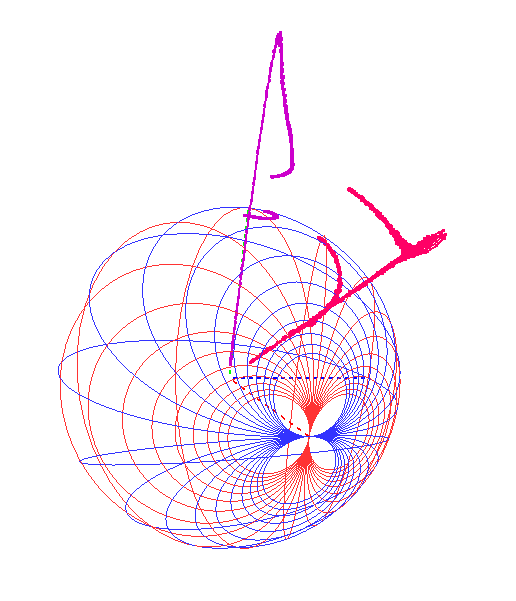
 **a b c d**


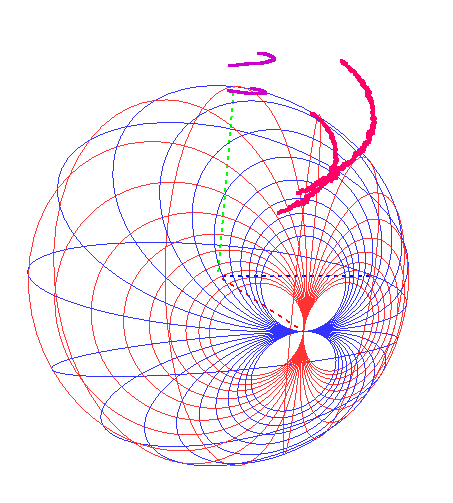

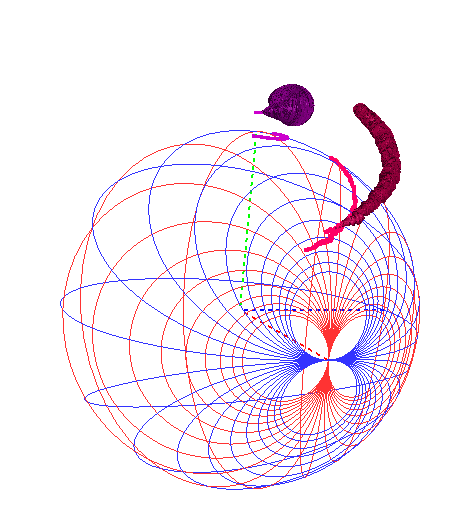

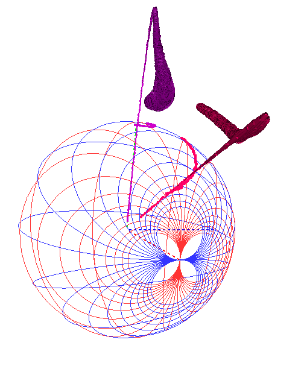


**Supplementary Fig. 20**: Comparative analyze of the new Peano inductor in the conductive state of VO_2_ versus previously reported reconfigurable inductor based on VO_2_ in the on state. (4GHz-15 GHz) on the 3D Smith chart: a, series inductance, b: shunt inductance, c: quality factor along the series inductance, d: quality factor along the shunt inductance. On the 3D smith chart one can see the S_11_ parameter to which they correspond.


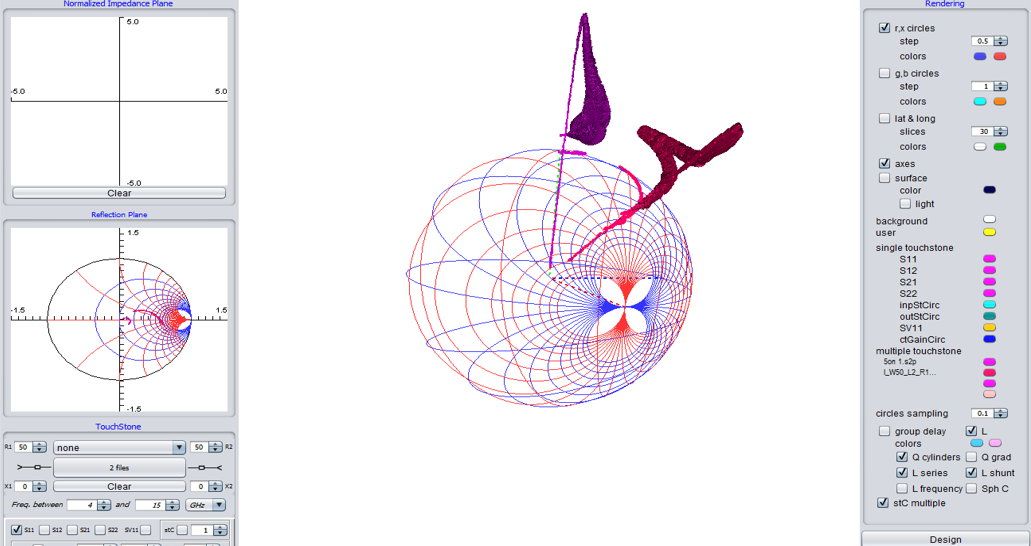


**Supplementary Fig. 21**: 3D Smith chart tool visualization of series and shunt inductances together with the quality factor on the 4-15 GHz frequency range.

Considering the simplified model in **Supplementary Fig. 15** it is important to stress that if R_S_=0 then the zeros of the imaginary part of Y_11_ are automatically zeros ( on the angular frequency axis) of the S_11_ parameter, since by using elementary ABCD matrices and one can easily determine the zeros of the Im (Y_11_) as:

$\boldsymbol{\omega}_{\boldsymbol{imy}\boldsymbol{11=}}\frac{\boldsymbol{1}}{\sqrt{\boldsymbol{L}_{\boldsymbol{s}}\boldsymbol{*}\boldsymbol{C}_{\boldsymbol{i}}\boldsymbol{+}\boldsymbol{L}_{\boldsymbol{S}}\boldsymbol{*}\boldsymbol{C}_{\boldsymbol{S}}}}$ (S46)

While the zeros (on the angular frequency axis) of the imaginary part of S_11_ include the zeros of the Y_11_

$\boldsymbol{\omega}_{\boldsymbol{imS}\boldsymbol{11=}}\frac{\boldsymbol{1}}{\sqrt{\boldsymbol{L}_{\boldsymbol{s}}\boldsymbol{*}\boldsymbol{C}_{\boldsymbol{i}}\boldsymbol{+}\boldsymbol{L}_{\boldsymbol{S}}\boldsymbol{*}\boldsymbol{C}_{\boldsymbol{S}}}}$ **,** $\frac{\sqrt{\boldsymbol{-L}_{\boldsymbol{s}}\boldsymbol{+2*}\boldsymbol{C}_{\boldsymbol{i}}\boldsymbol{*Z}}}{\sqrt{\boldsymbol{L}_{\boldsymbol{s}}\boldsymbol{*}\boldsymbol{C}_{\boldsymbol{i}}\boldsymbol{+}\boldsymbol{L}_{\boldsymbol{S}}\boldsymbol{*}\boldsymbol{C}_{\boldsymbol{S}}}}$ (S47)

**Our Peano inductors:**

A zoom of the VO_2_ switch of the fabricated inductor is shown in **Supplementary** **Fig. 22**. It is important to stress that our aim was to minimize resistive losses in the on state, thus we wished that the VO_2_ switch (gap in the Al at the top, where VO_2_ beneath is touched) should be as small in width as possible (while maintaining classical CMOS technology using standard microelectronic processes). This was desired since the conductivity of the VO_2_ in the on state is limited on SiO_2_/Si substrates.

**a**


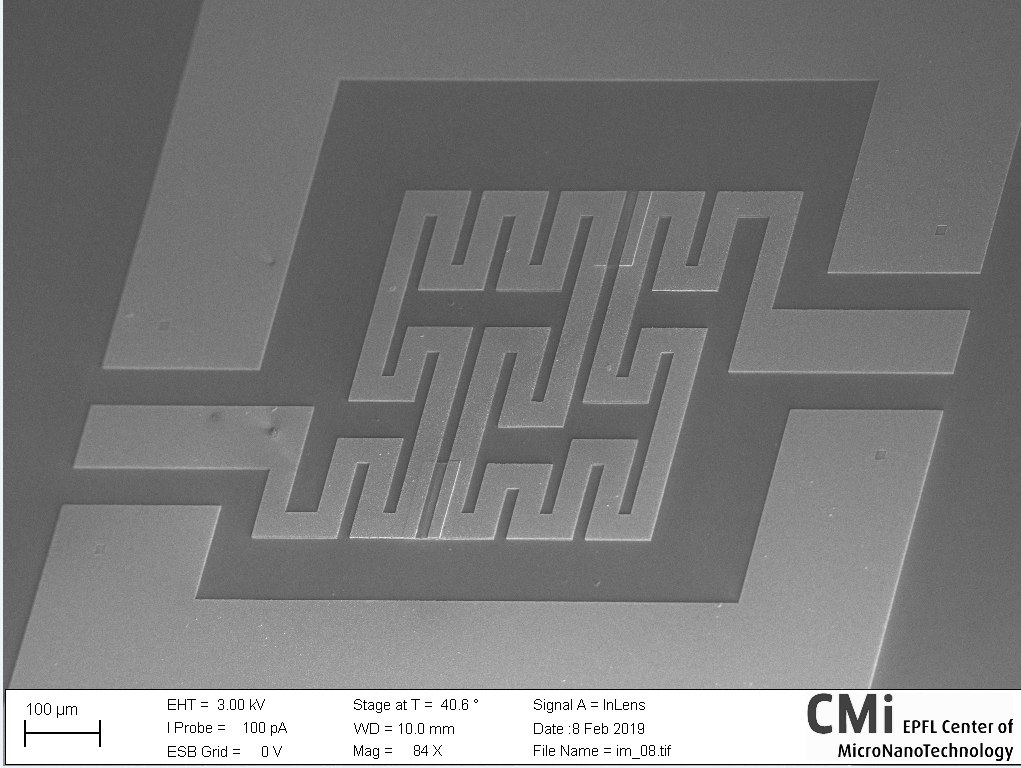


**b c**


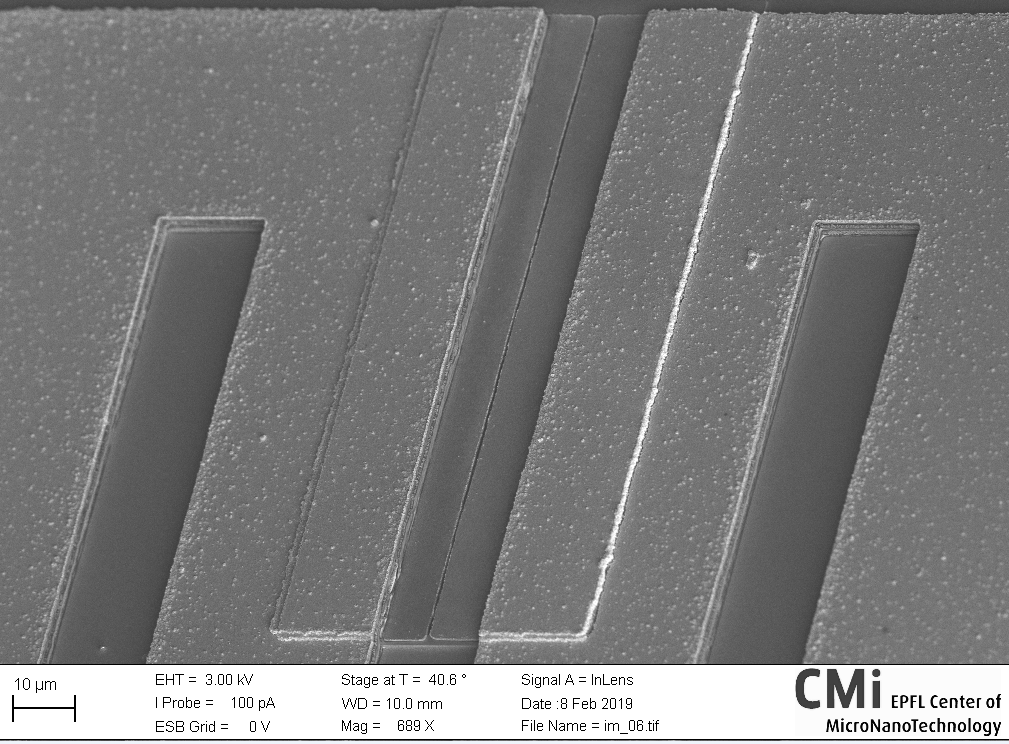

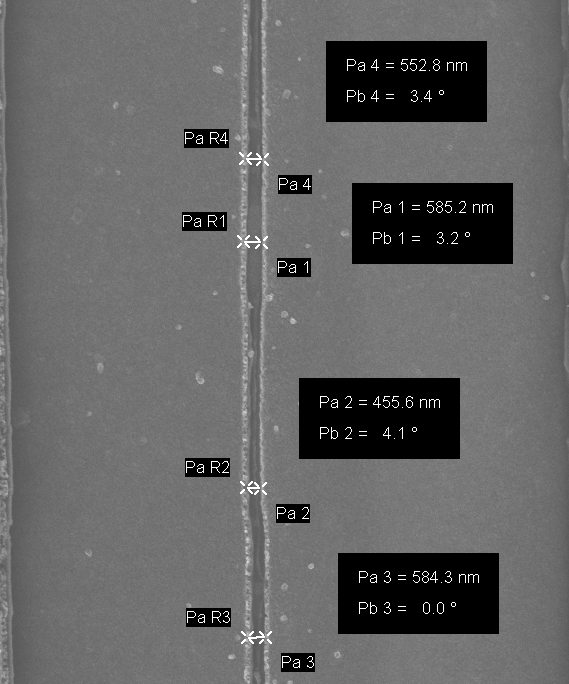


**Supplementary Fig. 22**: Peano fabricated inductor with a less than 600 nm gap in the Al connecting the VO_2_ thin film-2 switches.**a,** Inductor with two switches final layout.**b** switch fabricated layout. **c,** Zoom of the switch ( gap within the metallization where VO_2_ conductivity plays a key role)-in on state this gap plays a very important role-since it affects the losses ( the VO_2_ conductivity being below 49.000S/m) on SiO_2_/Si substrates. In off state the 21S/m conductivity of VO_2_ adds to dielectric losses of this material.

**References**

1. Tade, O.O., Gardner, P., Hall, S.S. Negative impedance converters for broadband antenna matching. In 2012 *42nd European Microwave Conference* (2012)*,* https://ieeexplore.ieee.org/document/6459295 (2012).
2. Tade, O.O., Gardner, P., Hall, S.S. Broadband matching of small antennas using negative impedance converters. In 2012 *IEEE International Symposium on Antenna and Propagation and USNC-URSI National Radio Science meeting* (2012). *https://ieeexplore.ieee.org/document/6348833*
3. Tade, O.O., Gardner, P., Hall, S.S. Antenna bandwidth broadening with a negative impedance converter. *Int. J. Microwave T*. **5** (3), 249-260 (2013).
4. Hrabar, S., Krois, I., Kiricenko, A. Towards active dispersion less ENZ metamaterial for cloaking applications. *Metamaterials*, **4**, 2-3, 89-97 (2010).
5. Goldstone, L. A low VSWR matching technique. (Correspondence and author’s reply). *IRE. Trans. Microw. Theory Tech*. **5**, 2, 163 (1957).
6. Munk, B.A. *Metamaterials: Critique and Alternatives*. John Wiley& Sons Inc., New Jersey, USA (2009).
7. Munk, B.A. *Finite Antenna Arrays and FSS.* John Wiley& Sons Inc., New Jersey, USA, (2003).
8. Best, S.R., The Foster reactance theorem and quality Factor for antennas. *IEEE Antennas Wireless Propag. Lett.* **3**, 306-309 (2004).
9. Berger, M., *A Panoramic View of Riemannian Geometry*. *Springer*. Berlin, 2003.
10. Do Carmo, M. P., *Differential Geometry of Curves and Surfaces*. Prentice-Hall inc., New Jersey, 1976.
11. Chen, M., Z., Q., A Note on Tests for Positive-Real Functions, *IEEE Trans. on Autom. Control*. **54**, 2, (2009).
12. Brune, O., Synthesis of a finite two-terminal network whose driving-point impedance is a prescribed function of frequency, PhD-thesis MIT, (1931). <https://onlinelibrary.wiley.com/doi/abs/10.1002/sapm1931101191>
13. Shi, T., Tang, M.-C., Wu, Z., Xu, H., Ziolkowski, R.W., Improved signal-to Noise Ratio (SNR), Bandwitdh-enhanced electrically small antenna augmented with Internal non-Foster elements. *IEEE Trans. Antennas Propag*. In press (2019) https://ieeexplore.ieee.org/stamp/stamp.jsp?tp=&arnumber=8624545&tag=1
14. Mirzaei, H., Eleftheriades, G., V., Realizing non-Foster reactive elements using negative-group-delay networks. *IEEE Trans. Microw. Theory Tech*., 61, 12, 4322–4332 (2013).
15. Muller, A.A., Lucyszyn, S., Properties of purely reactive Foster and Non-Foster passive networks. *IET Electron. Lett.,* **51**, 23, 1882-1884 (2015)
16. Bochert, M., *Infinite Regions of Various Geometries*. Bull. Amer. Math. Soc., **20**, 4, 185-200, (1914).
17. Brannan, D.A., Esplen, M.F., Gray, J.J., *Geometry*. Cambridge University Press, New York, 2007.
18. Muller, A.A, Soto, P., Dascalu, Boria V., The 3D Smith chart and its practical applications, *Microwave Journal*. **5**, 7, 64-74 (2012).
19. Muller, A. A., Soto, P. Dascalu, D, Neculoiu, D. Boria, V. E. A 3d Smith chart based on the Riemann sphere for active and passive microwave circuits. *IEEE Microw. and Wireless Comp. Lett.,* **21**, 6, 286-288 (2011).
20. Muller, A.A, et al. Apollonius unilateral transducer constant power gain circles on the 3D Smith chart”, *IET Electron. Lett.,* **50**, 21, 1531-1533 (2014).
21. Muller, A., A., Sanabria-Codesal, E., Moldoveanu, A. Asavei, V., Lucyszyn, S., Extended capabilities with group delay and resonator quality factor. *IEEE Trans. Microw. Theory Tech.,* **65**, 1, 10-17 (2017).
22. Muller, A. A., Moldoveanu A., Asavei V., Fleischer C. 3D Smith chart tool (2019) [www.3dsmithchart.com](http://www.3dsmithchart.com).
23. Smith, P.H. Transmission-line calculator. *Electronics* **12**, 29–31 (1939).
24. Zhu, B.O., Zhao J., Feng Y. Active impedance metasurface with full 360° reflection phase tuning. *Sci. Rep*. **3**, 3059 (2013).
25. Thang, B., Sodickson, D.K., Cloos, M.A. A high-impedance detector-array glove for magnetic resonance imaging of the hand. Nat. Biomed. Eng. **2**, 570-577 (2018).
26. Tuca, S.-S. et al. Calibrated complex impedance of CHO cells and E. coli bacteria at GHz frequencies using scanning microwave microscopy. *Nanotechnology* **27**, 135702 (2016).
27. Qu L., Zhang, Zhang R., Shin H., Kim J., Kim H. Mode-Controlled Wideband Slot-Fed Ground Radiation Antenna Utilizing Metal Loads for Mobile Applications. *IEEE Trans. Antennas Propag*. **65**, 2, 867-872 (2017).
28. Chu, H.C, Ma T. Beamwidth switchable planar microstrip series-fed slot array using reconfigurable synthesized transmission lines. *IEEE Trans. Antennas Propag*. **65**, 7, 3766-3771 (2017).
29. Liu, S., Liu A. A novel design methodology for high-efficiency current-mode and voltage-Mode class-E power amplifiers in wireless power transfer systems. *IEEE Trans. Power Electron*. 32, **6**, 4514-4521 (2017).
30. Ma, J., Steer, M. B., Jiang, X. An acoustic filter based on layered structure. Appl. Phys. Lett. **106**, 111903 (2015).
31. Dubois, M.-A., Murait P. Properties of aluminum nitride thin films for piezoelectric transducers and microwave filter applications. *Appl. Phys. Lett*. **74**, 20, 3032-3034 (1999).
32. Bochert, M. Infinite Regions of Various Geometries, *Bull. Amer. Math. Soc*., 20, 4,185-200 (1914).
33. Brannan, D.A., Esplen, M.F., Gray, J.J., Geometry, Cambridge University Press, New York (2007).
34. Casu, E.A. et al. A reconfigurable inductor based on Vanadium Dioxide insulator to metal transition. *IEEE Microw. Compon. Lett*. **29**, 9, 795-797 (2018).
35. Wang, S., Wang, W., Shin, E., Quach, Subramanyam, G. Tunable inductors using vanadium dioxide as the control material. *Microw. Opt. Techn. Lett.*, **59**, 5, 1057–1061 (2017).
36. Leroy, C. et al., High quality factor copper inductors integrated in deep dry-etched quartz substrates. *Microsyst. Technol.*, **13**, 11–12, 1483–1487 (2007).
37. Kang, J. et. al., On-chip intercalated-graphene inductors for next-generation radio frequency electronics. *Nature Electronics*, **1**, 46-51 (2018).
38. Wainstein, N., Kvatinsky, S. Time-tunable inductors using mermistors, *IEEE Trans. Circuits Syst. I, Reg. papers*, **65**, 5, 1505-1515 (2018).
39. Horng, T. S., Peng, K. C. , Jau,J.K, Tsai, Y.S. S-parameter formulation of quality factor for a spiral inductor in generalized two-port configuration. *IEEE Trans. Microw. Theory Tech.*, **51**, 11, 2197–2202 (2003).
